# Halogen bonding with carbon: directional assembly of non-derivatised aromatic carbon systems into robust supramolecular ladder architectures†

**DOI:** 10.1039/d3sc04191c

**Published:** 2023-10-24

**Authors:** Jogirdas Vainauskas, Tristan H. Borchers, Mihails Arhangelskis, Laura J. McCormick McPherson, Toni S. Spilfogel, Ehsan Hamzehpoor, Filip Topić, Simon J. Coles, Dmytro F. Perepichka, Christopher J. Barrett, Tomislav Friščić

**Affiliations:** a School of Chemistry, University of Birmingham Edgbaston Birmingham B15 2TT UK t.friscic@bham.ac.uk; b Department of Chemistry, McGill University 801 Sherbrooke St. W. H3A 0B8 Montreal Canada; c Faculty of Chemistry, University of Warsaw 1 Pasteura Street Warsaw 02-093 Poland; d EPSRC National Crystallography Service, School of Chemistry, University of Southampton, Highfield Southampton UK

## Abstract

Carbon, although the central element in organic chemistry, has been traditionally neglected as a target for directional supramolecular interactions. The design of supramolecular structures involving carbon-rich molecules, such as arene hydrocarbons, has been limited almost exclusively to non-directional π-stacking, or derivatisation with heteroatoms to introduce molecular assembly recognition sites. As a result, the predictable assembly of non-derivatised, carbon-only π-systems using directional non-covalent interactions remains an unsolved fundamental challenge of solid-state supramolecular chemistry. Here, we propose and validate a different paradigm for the reliable assembly of carbon-only aromatic systems into predictable supramolecular architectures: not through non-directional π-stacking, but *via* specific and directional halogen bonding. We present a systematic experimental, theoretical and database study of halogen bonds to carbon-only π-systems (C–I⋯π_C_ bonds), focusing on the synthesis and structural analysis of cocrystals with diversely-sized and -shaped non-derivatised arenes, from one-ring (benzene) to 15-ring (dicoronylene) polycyclic atomatic hydrocarbons (PAHs), and fullerene C_60_, along with theoretical calculations and a systematic analysis of the Cambridge Structural Database. This study establishes C–I⋯π_C_ bonds as directional interactions to arrange planar and curved carbon-only aromatic systems into predictable supramolecular motifs. In >90% of herein presented structures, the C–I⋯π_C_ bonds to PAHs lead to a general ladder motif, in which the arenes act as the rungs and halogen bond donors as the rails, establishing a unique example of a supramolecular synthon based on carbon-only molecules. Besides fundamental importance in the solid-state and supramolecular chemistry of arenes, this synthon enables access to materials with exciting properties based on simple, non-derivatised aromatic systems, as seen from large red and blue shifts in solid-state luminescence and room-temperature phosphorescence upon cocrystallisation.

## Abstract 2

Halogen bonds allow the directional assembly of robust supramolecular ladder architectures based on non-substituted carbon-only aromatic systems, providing an alternative to traditionally used non-directional π-stacking strategies.

## Graphical abstract

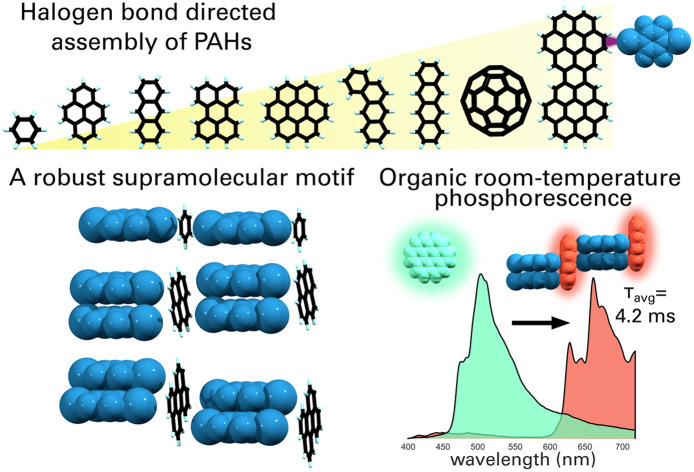

## Introduction

Polycyclic aromatic hydrocarbons (PAHs) are an extensive class of carbon-based molecules, estimated to hold 10–15% of all carbon in the known universe.^1–3^ PAH-based molecules are ubiquitous in the design of organic electronics^4,5^ and organic light emitting diodes (OLEDs).^6,7^ While the arrangement of PAH units in solids is of critical importance for their optical and electronic properties,^8^ controlling the assembly of PAHs in crystalline materials is a persistent challenge of organic solid-state chemistry.^9^ In most cases, guiding the arrangement of PAH units in the solid state requires derivatisation, either to introduce sterically demanding groups that modify molecular packing,^10^ or to introduce recognition sites for the formation of multi-component crystals (cocrystals) by directional interactions such as hydrogen (HB)^11–13^ or halogen bonding (XB).^14^

Such derivatisation strategies are, however, not applicable to the assembly of pristine, non-derivatised PAHs. Indeed, while carbon is central to organic chemistry, it is rarely considered a target of supramolecular recognition. Directional interactions such as HB^15^ and XB^16–18^ are generally regarded as insufficiently robust to enable predictable, directional assembly of carbon-only π-systems. As a result, approaches to molecular recognition, and in particular to cocrystallisation, of PAHs have traditionally been limited to non-directional face-to-face π–π stacking.^19^ This presents the design of supramolecular architectures based on directional assembly of carbon-only systems as an unsolved fundamental problem of solid-state supramolecular chemistry, and of the supramolecular chemistry of carbon in general.^20–22^

**Fig. 1:**
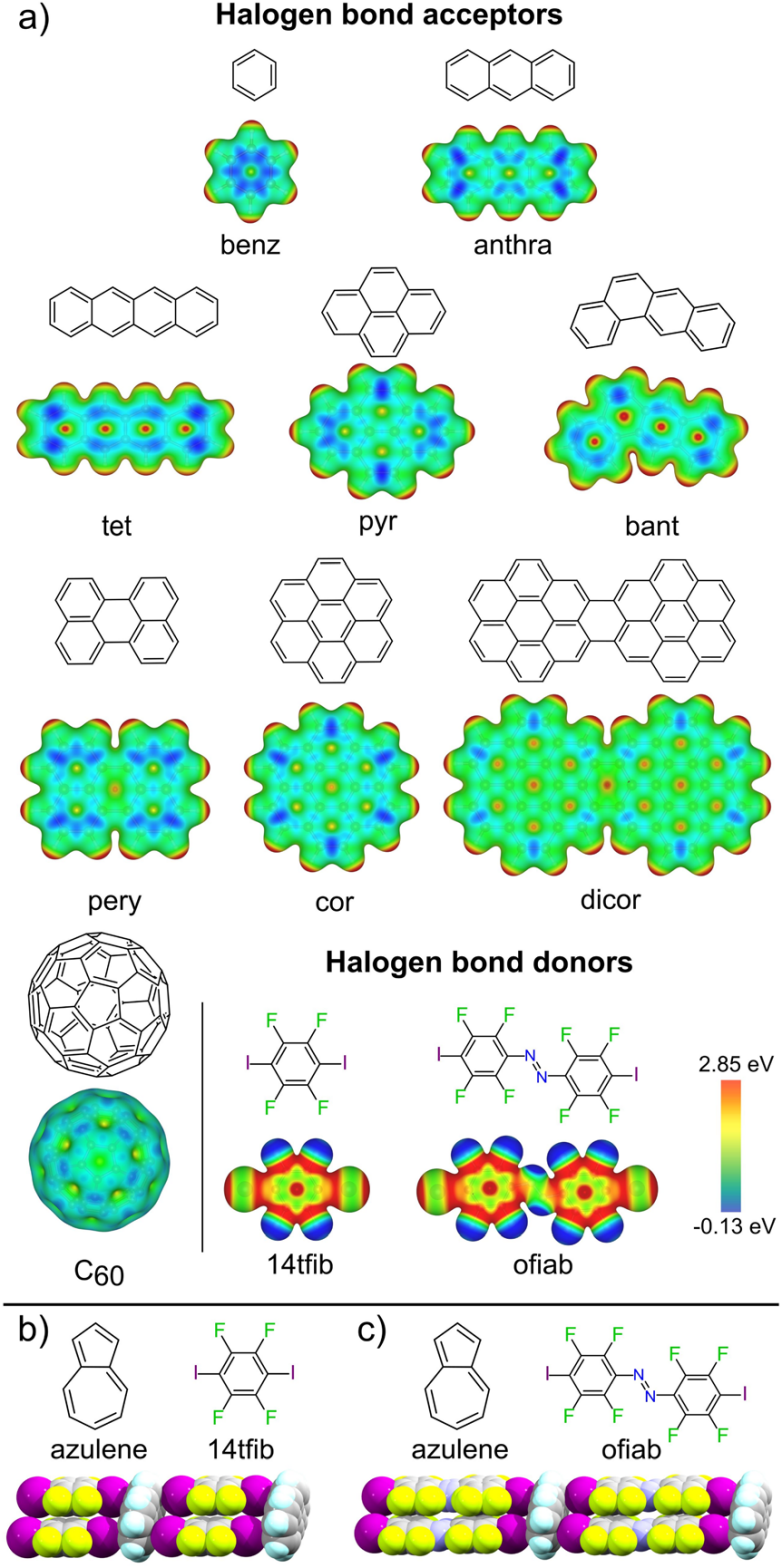
(a) Halogen bond acceptor and donor molecules explored in this work, along with corresponding electrostatic surface potential (ESP) maps, with isosurfaces plotted at 0.01 a.u. Fragments of the halogen-bonded C–I⋯π_C_ supramolecular ladder seen in the cocrystals of: (b) (azulene)(14tfib)_2_ and (c) (azulene)(ofiab)_2_ along with corresponding molecular diagrams.^43^

Halogen bonding has emerged as a versatile directional interaction in the crystalline solid state, offering access to a wider range of acceptor atoms than seen for hydrogen bonding.^23^ Scattered structural reports show that halogen bonds can form between electron-deficient iodine atoms as donors and flat, electron-rich π-systems, such as anilines, as acceptors.^24–34^ While cocrystals exhibiting individual XBs to carbon have been reported or theoretically studied, the possibility of using such interactions for the predictable assembly of supramolecular architectures based on PAHs has not been established.^35–42^ Recently,^43^ we observed that cocrystals of azulene with XB donors 1,4-diiodotetrafluorobenzene (tfib) or *trans*-octafluoro-4,4′-diiodoazobenzene (ofiab) (Fig. 1b and c) exhibit a ladder-like halogen-bonded motif involving aromatic carbon, in which the PAHs act as the rungs and the XB donors as rails, identical to the motif seen in a cocrystal of naphthalene^30^ with 14tfib. This unexpected observation of the same C–I⋯π_C_ ladder-like motif in three different cocrystals of naphthalene and azulene suggested an unexpected role for XB as a unique, possibly general tool for directional assembly of non-derivatised PAHs – contrasting the traditionally relied upon non-directional π-stacking.

**Fig. 2:**
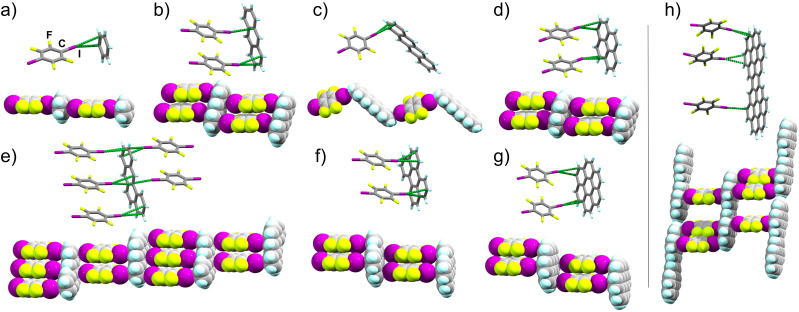
Stick model representation of halogen-bonded fragments in explored 14tfib cocrystals, with extended motifs shown using space-filling models: (a) (benz)(14tfib) (CCDC code 2240199), (b) (anthra)(14tfib)_2_ (CCDC code 2249511, also CSD JEJXOP),^55^ (c) (tet)(14tfib) (CCDC code 2240203), (d) (pyr)(14tfib)_2_ (CCDC code 2240200), (e) (bant)_2_(14tfib)_5_ (CCDC code 2281269), (f) (pery)(14tfib)_2_ (CCDC code 2240202), (g) (cor)(14tfib)_2_ (CCDC code 2240204), (h) (dicor)(14tfib)_3_ (CCDC code 2240207). The I⋯C_π_ intermolecular distances shorter than the sum of van der Waals radii for iodine and carbon atoms (3.68 Å) are shown as green dotted lines.^47^

We now provide an extensive and systematic study that demonstrates halogen bonding as a long-overlooked, reliable tool for the directional assembly of non-derivatised carbon-only aromatic systems. Our study, addressing diversely-shaped and -sized PAHs, as well as fullerene C_60_ (Fig. 1a), reveals the persistent formation of cocrystals based on a highly reproducible^44–46^ supramolecular ladder-like motif (Fig. 1b, c, and 2) of C–I⋯π_C_ halogen bonds. The robustness of this motif presents it as a unique example of a supramolecular synthon^47^ for the assembly of exclusively carbon-based aromatic systems through directional interactions, independent of molecular size, shape, or presence of π⋯π or C–H⋯π contacts. The presented set of cocrystals involving different combinations of 9 XB acceptors and 2 donors, along with theoretical calculations and a systematic analysis of the Cambridge Structural Database (CSD),^48^ establishes a different paradigm for the crystal engineering based on aromatic carbon: not *via* surface-based π-stacking, but by directional halogen bonding. The potential of this approach is evident by luminescence studies of select cocrystals based on pyrene, coronene and perylene, demonstrating extensive modification of emission properties due to halogen bond-directed assembly.

## Results and discussion

### Linear arenes (acenes)

Our first target was cocrystallisation of 14tfib with benzene (benz), the smallest possible arene that could be addressed in this study. Dissolving 14tfib in benz followed by slow evaporation produced large, colorless plate-like crystals. Removal of the crystals from the crystallisation vessel at room temperature, however, resulted in immediate degradation, evident by a loss of crystal transparency. Cooling the sample in dry ice enabled successful handling of the crystal and collection of X-ray single crystal diffraction data at 180 K (see ESI, Table S1†). Structure determination revealed that the crystals exhibit the composition (benz)(14tfib), based on one-dimensional (1D) supramolecular chains of alternating benz and 14tfib molecules, held by C–I⋯π_C_ halogen bonds between 14tfib iodine atoms and each face of benz molecules (Fig. 2a). Specifically, each 14tfib molecule forms multiple short I⋯π_C_ halogen-bonding contacts to the adjacent benz molecules, with intermolecular I⋯C distances (*d*_I⋯C_) of 3.48(2) Å and 3.51(2) Å, with the corresponding C–I⋯C angles (∠_C–I⋯C_) of 165° and 166°, respectively (Table 1, also ESI Table S2.†). The *d*_I⋯C_ separations are shorter than the expected sum of van der Waals radii (3.68 Å) for carbon and iodine.^49^

The simplicity of components makes it surprising that (benz)(14tfib) was not reported previously. The formation of halogen-bonded cocrystals with benz, however, is not without precedent, as 1D motifs similar to that in (benz)(14tfib) have been found with Br_2_ (ref. 50) and 1,4-diiodotetrachlorobenzene as XB donors.^51^ While the *d*_I⋯C_ distances in (benz)(14tfib) are ∼5% shorter than the sum of corresponding van der Waals radii, the shortest distance between the iodine atom of a 14tfib donor and a benz acceptor molecule is to the centroid of a carbon–carbon bond (3.42(1) Å). This is consistent with previously noted optimal geometry for halogen bonding to π-systems,^36,37^ and with the calculated electrostatic surface potential (ESP) surface of benz (Fig. 1a), showing that the areas of most negative ESP, expected to be beneficial for halogen bonding, are located on the arene π-bonds.

Bulk analysis of (benz)(14tfib) was hindered by rapid decomposition in air. Eventually, the formation of bulk cocrystal in a slurry of 14tfib in excess benz was confirmed by powder X-ray diffraction (PXRD) analysis of a sample under a plastic-wrap cover (see ESI, Fig. S1†).^52^ The (benz)(14tfib) structure confirms that the simplest aromatic hydrocarbon can be used as an acceptor in forming C–I⋯π_C_ chains (Fig. 2a) reminiscent of those in cocrystals of naphthalene and azulene with 14tfib or ofiab.^30,43^

Next, we targeted anthracene (anthra) and tetracene (tet) as XB acceptors. For anthra, a cocrystal of composition (anthra)(14tfib)_2_ was obtained by mechanochemical liquid-assisted grinding (LAG),^53,54^ using nitromethane as a liquid additive (ESI, Fig. S2†).^55^ Diffraction-quality single crystals of (anthra)(14tfib)_2_ were obtained by dissolving the mechanochemically made material in hot CH_2_Cl_2_, rapidly cooling in an ice bath for *ca.* 10 seconds, followed by slow evaporation. Structure analysis revealed that (anthra)(14tfib)_2_ is based on the ladder-like motif (Fig. 2b) with each face of the arene forming two C–I⋯π_C_ bonds with 14tfib molecules. The *d*_I⋯C_ distances ranged from 3.427(9) Å to 3.583(7) Å, with ∠_C–I⋯C_ angles in the range 157° to 173°. The distance between the iodine atom and the centroid of the relevant carbon–carbon bond was even shorter, at 3.366(5) Å, and no other short contacts were observed that would indicate C–H⋯π hydrogen bonding or π⋯π stacking between anthra molecules. The structure of (anthra)(14tfib)_2_ was also recently reported by Azzali *et al.* and our experimental data fully agrees with it.^55^

In the case of tet, the cocrystal (tet)(14tfib) was obtained in the form of diffraction-quality single crystals by slow evaporation of a solution of tet and 14tfib in 1,2,4-trichlorobenzene. Unlike the cocrystal containing anthra, the (tet)(14tfib) cocrystal does not exhibit the ladder-like motif, but instead presents a zig-zag architecture of alternating tet and 14tfib molecules interconnected *via* short, linear C–I⋯π_C_ contacts (Fig. 2c, Table 1 and ESI Table S2†). The individual I⋯C distances are again shorter than the sum of van der Waals radii for C and I atoms, at 3.410(6) Å and 3.509(7) Å, with respective ∠_C–I⋯C_ angles of 163° and 174°. The corresponding distance of the 14tfib iodine atom to the centroid of the relevant C–C bond on tet is again even shorter, at 3.387(5) Å, supporting the targeting of the π-system on the PAH. While tet is the only PAH in our study that did not produce the expected supramolecular ladder architecture, the (tet)(14tfib) structure confirms the general reliability of C–I⋯π_C_ halogen bonds for PAH cocrystallisation.

### Non-linear arenes

In order to explore the possibility of using XB for the assembly of arenes beyond the acene family, we targeted cocrystallisation with pyrene (pyr), benzanthracene (bant), perylene (pery) and coronene (cor), constituted of four, five and six fused ring systems. Mechanochemical screening in all cases produced new cocrystal phases, with compositions (pyr)(14tfib)_2_, (bant)_2_(14tfib)_5_, (pery)(14tfib)_2_ and (cor)(14tfib)_2_ (Fig. 2d–g). All four cocrystals were obtained in the form of diffraction-quality single crystals by crystallisation from CH_2_Cl_2_, and structural analysis revealed in each case the ladder motif analogous to the one seen with naphthalene,^30^ azulene,^43^ and anthra, with each face of the PAH participating in at least two linear C–I⋯π_C_ halogen bonds. The halogen-bonded distances were in each cocrystal comparable, in the range from 3.4 Å to 3.6 Å, with ∠_C–I⋯C_ angles in the range from 156° to 174° (see Table 1 and ESI Table S2†). Each cocrystal was also obtained mechanochemically as a bulk powder, exhibiting PXRD patterns consistent with herein determined crystal structures (see ESI†). The stability and composition of each cocrystal was validated by differential scanning calorimetry and thermogravimetric analysis (DSC and TGA, respectively, see ESI†). The DSC analysis revealed that the cocrystals were generally stable up to *ca.* 110 °C, and upon decomposition exhibited a weight loss step consistent with a loss of 14tfib. Structures of (pyr)(14tfib)_2_, (bant)_2_(14tfib)_5_, (pery)(14tfib)_2_, (cor)(14tfib)_2_ and (dicor)(14tfib)_3_ (Fig. 2d–h) further highlight the robustness of the supramolecular ladder motif based on C–I⋯π_C_ XB interactions, even with expanded aromatic systems as XB acceptors.

In the case of pyr, two other cocrystals with 14tfib have previously been reported, with different compositions: (pyr)(14tfib)^29^ and (pyr)_4_(14tfib).^56^ Both of these previously reported stoichiomorphs contain 14tfib molecules involved in either extended (for (pyr)(14tfib) cocrystal) or discrete (for (pyr)_4_(14tfib) cocrystal) arene-perfluoroarene π-stacking motifs with pyr molecules. The 14tfib molecules in these two prior cocrystals also form C–I⋯C halogen bonds of *ca.* 3.5 Å to neighboring pyr units. It is, therefore, notable, that the (pyr)(14tfib)_2_ phase reported here presents C–I⋯π_C_ halogen bonding as the dominant intermolecular interaction, without any notable herringbone C–H⋯π or arene-perfluoroarene π-stacking interactions seen in other stoichiomorphs (Fig. 2d, also see ESI Fig. S4†).^29,56^

Cocrystallisation of bant, an isomer of pyr and tet, with 14tfib resulted in a halogen-bonded cocrystal of composition (bant)_2_(14tfib)_5_. The cocrystal was found to again contain the anticipated ladder motif (Fig. 2e). The ladder motif in (bant)_2_(14tfib)_5_ is unique, however, with different faces of each bant unit alternatively taking part in either two or three C–I⋯π_C_ halogen bonds.

Crystal structures of cocrystals (pery)(14tfib)_2_ and (cor)(14tfib)_2_ (Fig. 2f and g) are, to the best of our knowledge, the first examples of halogen bonding being used to form cocrystals with large, non-substituted PAHs comprising >4 aromatic rings. To further examine the ability of C–I⋯π_C_ bonds to organise large arenes, we focused on dicoronylene (dicor), comprised of 15 aromatic rings (Fig. 1a). A sample of dicor was synthesised according to the reported procedure,^57^ by reaction of cor at 170 °C in a 3 : 1 by weight melt of AlCl_3_ and NaCl, followed by sublimation (see ESI†). Attempts to obtain cocrystals of 14tfib and dicor from common organic solvents were not successful due to poor arene solubility, with crystallisation eventually achieved from molten 14tfib at 110 °C. Single crystal X-ray analysis of the ruby-red crystals revealed a cocrystal structure with composition (dicor)(14tfib)_3_, exhibiting the C–I⋯π_C_ ladder motif, but with each face of the arene now engaged in XBs with 3 separate 14tfib donors (Fig. 2h). The XB distances lie in the range from 3.424(7) Å to 3.560(9) Å, with corresponding distances to the centroids of carbon–carbon bonds being in the range 3.327(6) Å to 3.579(8) Å. The halogen bonds exhibit a linear geometry, with ∠_C–I⋯C_ angles adopting values from 159–178°. Notably, the C–I⋯π_C_ bonds form on the edges of each dicor molecule, consistent with the calculated arene ESP surface, which shows that the highest negative potential is localised on the molecular rim. This means that a large fraction of the dicor molecule surface is not likely to engage in halogen bonding, and could be accessible for other types of interactions. Indeed, the dicor molecules are also engaged in π–π stacking between adjacent halogen-bonded ladders.

Of the three symmetrically distinct 14tfib molecules in the (dicor)(14tfib)_3_ structure, two also participate in F⋯F and C–H⋯F contacts^58^ with neighboring 14tfib and dicor molecules, while one 14tfib molecule also participates in F⋯I contacts.

The formation of this (dicor)(14tfib)_3_ shows that C–I⋯π_C_ halogen bonds are sufficiently robust to enable the cocrystallisation of large PAHs – to the best of our knowledge, no cocrystal of a non-derivatised PAH of similar size to dicor has ever previously been reported. Attempts to produce a bulk sample of (dicor)(14tfib)_3_ have so far been unsuccessful.

### Non-planar aromatic systems: C_60_

**Fig. 3:**
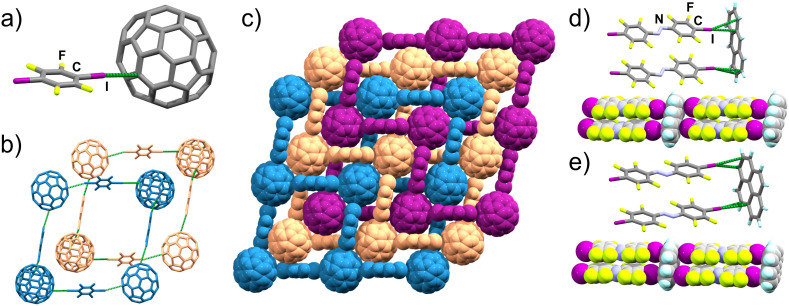
Structural motifs in (C_60_)(14tfib)_2_ and cocrystals of anthra and pyr with ofiab: (a) the C–I⋯C contact between iodine and C_60_ in (C_60_)(14tfib)_2_ (CCDC code 2240201); (b) interpenetration of two *sql*-grids in (C_60_)(14tfib)_2_; (c) space-filling representation of three distinct (C_60_)(14tfib)_2_*sql*-grids; (d) fragment of the supramolecular ladder in (anthra)(ofiab)_2_ (CCDC code 2240205) and (e) in (pyr)(ofiab)_2_ (CCDC code 2240206).

Next, we targeted a non-planar aromatic acceptor, buckminsterfullerene (C_60_). While previous work reported that C_60_ and 14tfib do not form a cocrystal,^39^ we found that milling of the two components produces a cocrystal of composition (C_60_)(14tfib)_2_. Dark-red block-shaped crystals of (C_60_)(14tfib)_2_ were subsequently obtained by slow evaporation of a toluene solution of the mechanochemically made material. Crystal structure analysis revealed that (C_60_)(14tfib)_2_ is based on interpenetrated square-grid topology (*sql*) nets of C–I⋯π_C_ bonds (Fig. 3a) with *d*_I⋯C_ separation of 3.54(1) Å and halogen bond angle of 163°. The *sql*-nets are formed by C_60_ molecules acting as four-fold nodes with 14tfib molecules as framework linkers (Fig. 3b and c). The structure also exhibits short C⋯F contacts between 14tfib and C_60_ molecules in adjacent nets (*d*_F⋯C_ = 3.17(1) Å; ∠_C–F⋯C_ = 102.8(4)°). Notably, nearest-neighbor C_60_ molecules do not exhibit any short mutual contacts, and at room temperature do not exhibit the rotational disorder commonly seen in C_60_-containing crystal structures.^59–62^ Analysis of (C_60_)(14tfib)_2_ by DSC and TGA reveals decomposition around 110 °C, evidenced by a single endothermic event in the DSC thermogram, accompanied by the loss of 50% of sample weight, consistent with the theoretical content of 14tfib (52.7%).

### Replacement of the halogen bond donor

In order to further explore the generality of the supramolecular ladder motif formed with PAHs, we next attempted cocrystallisation of anthra and pyr with ofiab, a longer XB donor. In each case, milling of the XB donor and acceptor followed by PXRD analysis revealed a new cocrystal phase, with compositions (anthra)(ofiab)_2_ and (pyr)(ofiab)_2_. Crystallographic analysis of single crystals grown by recrystallisation of the mechanochemically-made material from CH_2_Cl_2_ revealed the expected C–I⋯π_C_ ladder-like architectures in both cases (Fig. 3d, e, Table 1 and ESI Table S2†). Importantly, the measured I⋯C and I···centroid distances are not appreciably different from those in (anthra)(14tfib)_2_ and (pyr)(14tfib)_2_. Both (anthra)(ofiab)_2_ and (pyr)(ofiab)_2_ were found to melt around 160–170 °C, as established by DSC, with TGA showing that samples completely evaporate, in a single step, at temperatures around 200 °C.

### Periodic DFT calculations

The formation of herein reported cocrystals was also investigated by plane-wave periodic-density functional theory (DFT) calculations in CASTEP,^63^ using PBE^64^ functionals combined with Grimme D3 semiempirical dispersion correction.^65^ Periodic DFT was previously shown^66^ to be effective for understanding the thermodynamic stability of halogen-bonded cocrystals. The calculated cocrystal formation energies (*E*_calc_) with respect to individual crystalline components (Table 1) were found to be negative in all but one case, indicating that cocrystallisation was enthalpically favorable in general. The exception is (dicor)(14tfib)_3_, with *E*_calc_ of +6.48 kJ mol^−1^ suggesting that its formation may be entropy driven. Vibrational contributions to the Free energies related to cocrystallisation were, however, not taken into account due to the high computational cost of periodic DFT phonon calculations.

**Table 1:** Calculated energies of cocrystal formation (*E*_calc_, in kJ mol^−1^) from starting materials, halogen bond interaction energies (*E*_XB_, in kJ mol^−1^), and experimental C–I⋯C (in Å) distances for herein reported cocrystals.^a^ For structures where an arene molecules forms C–I⋯π_C_ halogen bonds with multiple XB donors, the average *E*_XB_ is provided, with individual *E*_XB_ values in brackets

Cocrystal	*E*_calc_/kJ mol^−1^	*E*_XB_/kJ mol^−1^	*d*(C–I⋯C_π_)/Å
(benz)(14tfib)	−10.4^b^	−16.43	3.48(2), 3.51(2)
(anthra)(14tfib)_2_	−5.89	−18.82 (−19.28, −18.36)	3.427(9), 3.445(8), 3.583(7)
(tet)(14tfib)	−2.27	−13.76	3.410(6), 3.509(7)
(pyr)(14tfib)_2_	−5.90	−18.86 (−18.63, −19.10)	3.515(8), 3.56(1), 3.50(1), 3.50(1)
(bant)_2_(14tfib)_5_	−5.54	−19.48 (−19.13, −20.29, −18.30, −17.45, −22.25)	3.50(2), 3.64(2), 3.50(1), 3.46(1), 3.51(3), 3.39(2), 3.48(2), 3.40(2), 3.55(1)
(pery)(14tfib)_2_	−8.46	−20.45 (−20.45, −20.45)	3.509(9), 3.430(8), 3.482(9), 3.51(1)
(cor)(14tfib)_2_	−5.93	−20.45 (−20.45, −20.45)	3.45(1), 3.46(2), 3.46(1), 3.51(2)
(dicor)(14tfib)_3_	6.48	−20.12 (−19.54, −20.29, −20.55)	3.440(8), 3.424(7), 3.368(8), 3.38(1), 3.560(9)
(anthra)(ofiab)_2_	−2.91	−19.66 (−19.30, −20.31)	3.62(2), 3.43(1), 3.55(2), 3.63(2)
(pyr)(ofiab)_2_	−0.17	−20.33 (−20.00, −20.66)	3.476(7), 3.394(7), 3.586(7), 3.448(6)
(C_60_)(14tfib)_2_	−13.95	−15.15	3.54(1)

a The full set of XB bonds and angles is provided in ESI Table S2.

b Calculation based on the orthorhombic polymorph of benz (CSD code BENZEN).

The energies associated with individual halogen bonds (*E*_XB_) were obtained by subtracting the total energies of individual component molecules from the total energy of each distinct XB donor–acceptor dimer unit found in the cocrystal structure. The *E*_XB_ values were highly consistent across all explored XB acceptors, with all dimer interaction energies being exothermic and falling within the range of −13 kJ mol^−1^ to −21 kJ mol^−1^.

### Analysis of the Cambridge structural database (CSD)

Our systematic experimental study reveals the robust supramolecular synthon based on C–I⋯π_C_ halogen bonds for the directional assembly of carbon-only aromatic systems of various shapes and sizes. It is highly surprising that this expansive potential for directional arene assembly by C–I⋯π_C_ bonds has not previously been noted, leading us to conduct an in-depth search of the Cambridge Structural Database (CSD) to probe for the appearance and geometry of such interactions involving the iodine atom of a C–I fragment as a donor and a carbon atom of a 5-, 6- or a 7-membered ring, a C=C, or a C≡C moiety, as the acceptor. As XBs are expected to be shorter than the sum of the van der Waals radii of interacting atoms, the search was limited to I⋯C distances up to 4.68 Å, which is *ca.* 1 Å longer than the sum of van der Waals radii for carbon (1.7 Å) and iodine (1.98 Å).^49^

**Fig. 4:**
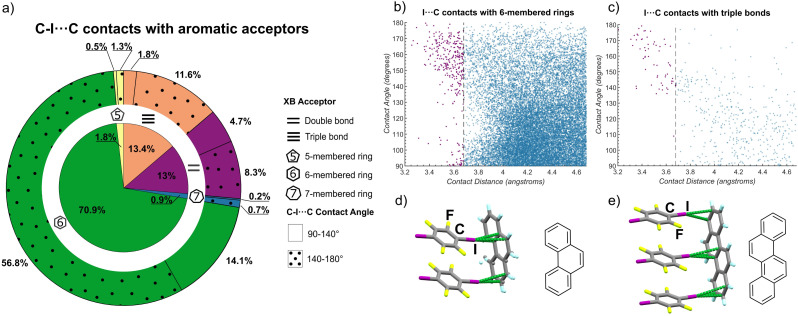
Outcomes of CSD searches for short C–I⋯C contacts to alkene, alkyne, 5-, 6- and 7-membered ring acceptors based on aromatic carbon and selected representative structures. (a) Distribution of the I⋯C contact lengths shorter than 4.68 Å across different acceptor types. For each XB acceptor type, the fraction of contacts with a linear geometry (C–I⋯C angle in the range from 140° to 180°) corresponding to halogen bonding, is indicated on the outer circle by a dotted pattern. Plots of the distribution of lengths and angles for C–I⋯C contacts to: (b) 6-membered ring and (c) alkyne (C≡C) acceptors. To guide the eye, contacts shorter than the van der Waals limit of 3.68 Å are shown in purple. Plots of the distribution of C–I⋯C contact lengths and angles for 5- and 7-membered ring, as well as alkene (C=C) acceptors, are given in the ESI.† Fragment of the crystal structures^67^ of: (d) (phenanthrene)(14tfib)_2_ and (e) (chrysene)(14tfib)_3_, illustrating the appearance of the C–I⋯π_C_ ladder motif.

The CSD searches revealed that short contacts between iodine and 6-membered rings are the most prevalent, representing *ca.* 71% of the total short contacts examined, followed by contacts to C≡C bonds (13%), C=C bonds (13%), while contacts involving 5- and 7-membered rings represented *ca.* 2% and 1% of all identified short contacts (Fig. 4a). The CSD searches indicated two geometry-distinct regimes of C–I⋯π_C_ interactions, dependent on the I⋯C_π_ distance. Specifically, there was no particular geometrical preference for longer I⋯C separations. However, shorter I⋯C distances were generally associated with a preference for linear C–I⋯C contacts, with angles in the range of *ca.* 140–180°. Such behavior was particularly pronounced for C≡C bonds and 6-membered rings as acceptors, where the majority of I⋯C distances below ∼3.7 Å were associated with a C–I⋯C angle of 140° or higher (Fig. 4b and c). Such preference for linear geometries at lower I⋯C distances is consistent with XBs,^14*d*^ reinforcing the herein proposed role of halogen bonds as an overlooked, yet consistent, strategy for directional assembly of carbon-based aromatic system, and suggest the possible existence of other C–I⋯π_C_ driven supramolecular synthons. In addition to structures identified in the CSD, during preparation of this manuscript two crystal structures were reported^67^ that strongly reinforce the view of XBs as reliable interactions to form supramolecular architectures based on PAHs (Fig. 4d and e). These structures are based on phenanthrene and on chrysene, an isomer of tet and bant, and exhibit the C–I⋯π_C_ ladder motif with PAH as the XB acceptor and 14tfib as the donor.

### Luminescence properties

Organic materials with emissive triplet states and long emission lifetimes (phosphorescence) are being sought for applications in OLEDs,^68^ biological imaging,^69^ anticounterfeiting^70^ and more.^71^ However, purely organic systems rarely exhibit phosphorescence at room temperature, due to the spin-forbidden nature of the singlet-to-triplet transition.^72–74^ Recent work shows the critical roles of intermolecular interactions and crystal packing on organic room temperature phosphorescence efficiency.^74–76^ In this context, cocrystallisation of PAHs offers a promising platform for achieving organic phosphorescent materials, as the solid-state arrangement of aromatic units directly influences diverse optoelectronic properties, such as excitation/emission wavelengths, and lifetime kinetics.^77,78^ Halogen bonded arene cocrystals have been reported to exhibit phosphorescence decays on the scale of several milliseconds,^27–32,42,67,77^ with an isolated case of decays >200 ms.^77*b*^ Recently, Abe *et al.* reported luminescence properties of a (phenanthrene)(14tfib)_2_ cocrystal, and found that partial replacement (up to 25%) of phenanthrene with pyr leads to an increase of the phosphorescence quantum yield from *ca*. 6 to >20%.^67^ Further replacement of phenanthrene with pyr (up to 50%), however, led to a drop of phosphorescence efficiency. Notably, a cocrystal of composition (pyr)(14tfib)_2_, in which all phenanthrene would be replaced by pyr, was not reported – but (pyr)(14tfib) showed a low luminescence quantum yield <1% and short lifetime of 50 μs^67^ (500 μs^29^).

**Fig. 5:**
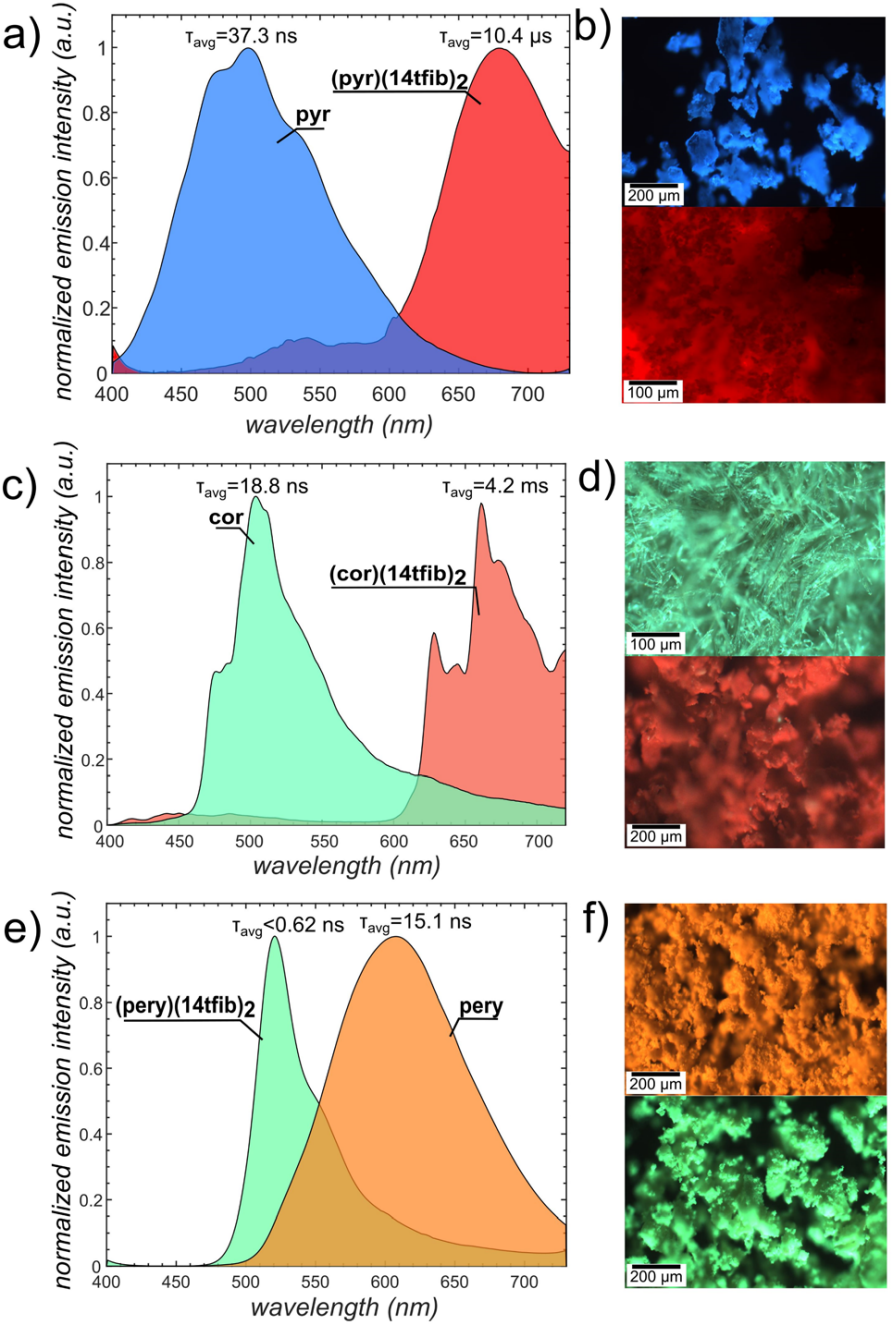
Change in luminescence spectra upon the formation of halogen-bonded cocrystals with aromatic XB acceptors. (a) Solid-state luminescence spectra for pyr (blue) and (pyr)(14tfib)_2_ (red). (b) A sample of (top) pyr and (bottom) (pyr)(14tfib)_2_ excited with 365 nm LED light. (c) Solid-state luminescence spectra for cor (green) and (cor)(14tfib)_2_ (red). (d) A sample of (top) cor and (bottom) (cor)(14tfib)_2_ excited with 365 nm LED light. (e) Solid-state luminescence spectra for α-pery and (pery)(14tfib)_2_. (f) A sample of (top) α-pery and (bottom) (pery)(14tfib)_2_ excited with 365 nm LED light.

Having herein synthesised the previously missing cocrystal of composition (pyr)(14tfib)_2_, we explored its photoluminescence properties, along with those of the heavier congeners (pery)(14tfib)_2_ and (cor)(14tfib)_2_ (Fig. 5). The cocrystals of cor, pyr, or pery with 14tfib all exhibited strong luminescence shifts when compared to the pristine solid PAH. The previously not accessible (pyr)(14tfib)_2_ exhibited a remarkable bathochromic shift of almost 200 nm, with the maximum emission wavelength (*λ*_max_) at 678 nm (red), compared to 498 nm (blue) for solid pyr (Fig. 5a and b). The red-shifted *λ*_max_ and the emission life-time (10.4 μs) are similar to the previously reported behavior of (pyr)(14tfib)^29,67^ and suggest the phosphorescent nature of the emission. A similar behavior was displayed by (cor)(14tfib)_2_ cocrystals: a significant ∼150 nm red shift of *λ*_max_ (from 513 nm in pristine solid cor to 660 nm in the cocrystal, Fig. 5c and d) and very long emission life-time (4.2 ms). Notably, the emission of (cor)(14tfib)_2_ extends in the NIR region, beyond 750 nm, which is typically difficult to achieve because of the energy-gap law.^78^

In a sharp contrast to the red-shifted emission (pyr)(14tfib)_2_ and (cor)(14tfib)_2_ cocrystals, the (pery)(14tfib)_2_ cocrystals exhibited a strong ∼100 nm hypsochromic shift of emission, from *λ*_max_ = 607 nm (orange) in pristine pery solid (α-pery polymorph) to 521 nm (green) of the cocrystal (Fig. 5e and f). Such blue-shifting behavior is unusual, as cocrystals exhibiting C–I⋯π_C_ interactions generally exhibit red-shifted emission compared to the pure emissive component solid.^79^ This behavior of (pery)(14tfib)_2_ is reminiscent of the J-aggregate emission of the β-pery crystals^80^ in which the pery molecules are arranged in off-diagonal interaggregate interactions, similar to (pery)(14tfib)_2_ (see ESI, Fig. S36†). Such J-aggregated states are known to enhance the fluorescence emission radiative rates, outcompeting intersystem crossing rates from populating triplet excitons.^81^ Indeed, the emission lifetime observed in (pery)(14tfib)_2_ was <0.62 ns (see ESI, Fig. S35†). The observed blue shift of (pery)(14tfib)_2_ cocrystals (as well as β-pery crystals) compared to α-pery is explained by disruption of the excimer emission resulting from the π-dimer packing motif of the latter structure.

Consequently, the incorporation of the C–I⋯π_C_ interactions in co-crystals of the PAHs can enable their room-temperature phosphorescence (RTP) through the heavy atom effect, and is commonly manifested in the red-shifted emission of cocrystals compared to pure PAH. However, the RTP properties also depend on a balance of inter-system crossing *vs.* fluorescence transition which varies with the molecular and crystal structure. Thus, the fast fluorescence transition in (pery)(14tfib)_2_ brought about by the specific packing of PAH molecules (J-aggregation) prevents the observation of RTP.

## Conclusions

In summary, we have presented an extensive and systematic experimental, theoretical, and database study that establishes halogen bonding to aromatic carbon systems as a powerful, but historically overlooked approach for the design of supramolecular architectures based on carbon as the molecular recognition site. This work coalesces and advances scattered reports of halogen bonds to π-systems, and demonstrates not only that C–I⋯π_C_ halogen bonding can be reliably used as a directional interaction for the synthesis of cocrystals of differently-sized and -shaped carbon-only aromatic systems, but also establishes the supramolecular ladder structure based on C–I⋯π_C_ halogen bonds as a unique supramolecular synthon generally applicable to non-substituted carbon systems.

In particular, C–I⋯π_C_ halogen bonding has been effective in cocrystallisation of all 9 explored aromatic carbon molecules, from benzene (one aromatic ring) to dicoronylene (15 fused aromatic rings), resulting in altogether 11 cocrystals. Focusing on PAHs only (*i.e.*, excluding benzene and C_60_), C–I⋯π_C_ halogen bonding led to the formation of the expected supramolecular ladder motif in 8 out of 9 cases, with PAH units acting as the rungs and halogen bond donor molecules as the rails. The reliability of a self-assembly motif can be evaluated through Aakeröy's concept of supramolecular yield.^44–46^ From that perspective, the synthesis of C–I⋯π_C_ ladder motifs based on non-substituted arene hydrocarbons is highly reliable, with an 89% supramolecular yield. If one includes in this overview the recently reported cocrystals of naphthalene,^30^ azulene (two cocrystals),^43^ phenanthrene and chrysene,^67^ which were all found to exhibit the C–I⋯π_C_ ladder motif, the supramolecular yield rises to 13 out of 14 cases, *i.e.* 93%.

The high reliability of this supramolecular cocrystal-forming reaction is surprising when considering that traditionally the only reliable way to achieve cocrystallisation of arenes was through π-stacking. The C–I⋯π_C_ halogen-bonded ladder now suggests a route for crystal engineers to rationally use non-substituted PAHs as building blocks in designing new carbon-based supramolecular architectures, different from π-stacked arrays.‡‡d'Agostino and co-workers^42^ have reported that the aromatic hydrocarbons stilbene and tolane form halogen-bonded cocrystals with 14tfib which, although they do not exhibit the C–I⋯π_C_ ladder synthon, are based on a closely-related sheet architecture. The C–I⋯π_C_ halogen bonds underlying the ladder motif often coincide with the sites of greatest negative ESP on the arene, and a single side of a six-membered carbon ring is never observed to participate in more than one halogen bond. These observations establish a different element of design for the assembly of supramolecular architectures from non-substituted arenes, based on the directional interaction of halogen bonding, rather than the traditionally used and non-directional π-stacking. The ability to generate new structures based on simple PAH components is of significance in the context of materials with new properties. That is illustrated by very large red or blue shifts (in the range of *ca.* 100 nm to 200 nm), as well as room-temperature phosphorescence achieved for PAHs engaged in the supramolecular C–I⋯π_C_ ladder structures. In particular, the phosphorescence lifetime of >4 ms observed for the coronene-based cocrystal provides further incentive to explore the design of fully organic phosphorescent systems based on halogen bonding to aromatic system.

## Data availability

Data supporting this manuscript has been provided as ESI.† Selected data relevant to PXRD analysis, fluorescence emission spectroscopy, and theoretical modelling, is also available from the data repository Zenodo (DOI: https://doi.org/10.5281/zenodo.10064165). Crystallographic data in CIF format has been deposited with the Cambridge Structural Database (CCDC 2240199–2240207, 2249511 and 2281269).

## Author contributions

The manuscript was written through contributions of all authors. All authors have given approval to the final version of the manuscript.

## Conflicts of interest

There are no conflicts to declare.

## Supplementary Material

SC-014-D3SC04191C-s001

SC-014-D3SC04191C-s002

SC-014-D3SC04191C-s003

## References

[cit1] Peeters E. and Cami J., in Encyclopedia of Astrobiology, ed. M. Gargaud, R. Amils, J. C. Quintanilla, H. J. Cleaves, W. M. Irvine, D. L. Pinti and M. Viso, Springer Berlin Heidelberg, Berlin, Heidelberg, 2011, ch. 1250, pp. 1307–1321

[cit2] Peeters E., Mackie C., Candian A., Tielens A. G. G. M. (2021). Acc. Chem. Res..

[cit3] Hansen C. S., Peeters E., Cami J., Schmidt T. W. (2022). Commun. Chem..

[cit4] Aumaitre C., Morin J. F. (2019). Chem. Rec..

[cit5] Zaumseil J., Sirringhaus H. (2007). Chem. Rev..

[cit6] Lee J. H., Chen C. H., Lee P. H., Lin H. Y., Leung M. K., Chiu T. L., Lin C. F. (2019). J. Mater. Chem. C.

[cit7] Liang N., Zhao Y. K., Wu Y. Z., Zhang C. R., Shao M. (2021). Appl. Phys. Lett..

[cit8] Yang X., Lan L., Pan X., Liu X., Song Y., Yang X., Dong Q., Li L., Naumov P., Zhang H. (2022). Nat. Commun..

[cit9] Yu P. P., Zhen Y. G., Dong H. L., Hu W. P. (2019). Chem.

[cit10] Anthony J. E. (2006). Chem. Rev..

[cit11] Gupta P., Karothu D. P., Ahmed E., Naumov P., Nath N. K. (2018). Angew. Chem., Int. Ed..

[cit12] Yelgaonkar S. P., Campillo-Alvarado G., MacGillivray L. R. (2020). J. Am. Chem. Soc..

[cit13] Krishna G. R., Devarapalli R., Lal G., Reddy C. M. (2016). J. Am. Chem. Soc..

[cit14] Aakeröy C. B., Baldrighi M., Desper J., Metrangolo P., Resnati G. (2013). Chem.–Eur. J..

[cit15] Ferguson G., Gallagher J. F., Glidewell C., Zakaria C. M. (1994). Acta Crystallogr., Sect. C: Struct. Chem..

[cit16] Geiser U., Kumar S. K., Savall B. M., Harried S. S., Carlson K. D., Mobley P. R., Wang H. H., Williams J. M., Botto R. E. (1992). Chem. Mater..

[cit17] Goud N. R., Bolton O., Burgess E. C., Matzger A. J. (2016). Cryst. Growth Des..

[cit18] Cao D., Hong M., Blackburn A. K., Liu Z., Holcroft J. M., Stoddart J. F. (2014). Chem. Sci..

[cit19] Cho H. J., Kim S. W., Kim S., Lee S., Lee J., Cho Y., Lee Y., Lee T.-W., Shin H.-J., Song C. (2020). J. Mater. Chem. C.

[cit20] MacGillivray L. R. (2004). CrystEngComm.

[cit21] Gunawardana C. A., Aakeröy C. B. (2018). Chem. Commun..

[cit22] Nangia A. K., Desiraju G. R. (2019). Angew. Chem., Int. Ed..

[cit23] Cinčić D., Friščić T., Jones W. (2008). J. Am. Chem. Soc..

[cit24] Ding X. H., Chang Y. Z., Ou C. J., Lin J. Y., Xie L. H., Huang W. (2020). Natl. Sci. Rev..

[cit25] Sharber S. A., Mullin W. J., Thomas S. W. (2021). Chem. Mater..

[cit26] Liantonio R., Luzzati S., Metrangolo P., Pilati T., Resnati G. (2002). Tetrahedron.

[cit27] Wang W. Z., Zhang Y., Jin W. J. (2020). Coord. Chem. Rev..

[cit28] Li L. L., Liu Z. F., Wu W. X., Jin W. J. (2018). Acta Crystallogr., Sect. B: Struct. Sci., Cryst. Eng. Mater..

[cit29] Shen Q. J., Wei H. Q., Zou W. S., Sun H. L., Jin W. J. (2012). CrystEngComm.

[cit30] Shen Q. J., Pang X., Zhao X. R., Gao H. Y., Sun H.-L., Jin W. J. (2012). CrystEngComm.

[cit31] Gao H. Y., Zhao X. R., Wang H., Pang X., Jin W. J. (2012). Cryst. Growth Des..

[cit32] Gao H. Y., Shen Q. J., Zhao X. R., Yan X. Q., Pang X., Jin W. J. (2012). J. Mater. Chem..

[cit33] Li L., Wang H., Wang W., Jin W. J. (2017). CrystEngComm.

[cit34] Li L., Wu W. X., Liu Z. F., Jin W. J. (2018). New J. Chem..

[cit35] Ang S. J., Mak A. M., Sullivan M. B., Wong M. W. (2018). Phys. Chem. Chem. Phys..

[cit36] Forni A., Pieraccini S., Rendine S., Gabas F., Sironi M. (2012). ChemPhysChem.

[cit37] Kim D. Y., Madridejos J. M. L., Ha M., Kim J. H., Yang D. C., Baig C., Kim K. S. (2017). Chem. Commun..

[cit38] Mooibroek T. J., Gamez P. (2013). CrystEngComm.

[cit39] Zhang Y., Wang J. G., Sun X., Liu Q., Wang W., Wang Y. B. (2018). ChemPlusChem.

[cit40] Mikherdov A. S., Novikov A. S., Boyarskiy V. P., Kukushkin V. Y. (2020). Nat. Commun..

[cit41] Mikherdov A. S., Popov R. A., Smirnov A. S., Eliseeva A. A., Novikov A. S., Boyarskiy V. P., Gomila R. M., Frontera A., Kukushkin V. Y., Bokach N. A. (2022). Cryst. Growth Des..

[cit42] d'Agostino S., Grepioni F., Braga D., Ventura B. (2015). Cryst. Growth Des..

[cit43] Vainauskas J., Topić F., Bushuyev O. S., Barrett C. J., Friščić T. (2020). Chem. Commun..

[cit44] Aakeröy C. B., Beatty A. M., Helfrich B. A. (2002). J. Am. Chem. Soc..

[cit45] Aakeröy C. B., Beatty A. M., Helfrich B. A. (2001). Angew Chem. Int. Ed. Engl..

[cit46] Aakeröy C. B., Salmon D. J. (2005). CrystEngComm.

[cit47] Desiraju G. R. (1995). Angew Chem. Int. Ed. Engl..

[cit48] Groom C. R., Bruno I. J., Lightfoot M. P., Ward S. C. (2016). Acta Crystallogr., Sect. B: Struct. Sci., Cryst. Eng. Mater..

[cit49] Mantina M., Chamberlin A. C., Valero R., Cramer C. J., Truhlar D. G. (2009). J. Phys. Chem. A.

[cit50] Hassel O., Strømme K. O., Haraldsen H., Grönvall A., Zaar B., Diczfalusy E. (1958). Acta Chem. Scand..

[cit51] Bosch E. (2019). IUCrData.

[cit52] Huskić I., Christopherson J. C., Užarević K., Friščić T. (2016). Chem. Commun..

[cit53] Friščić T., Childs S. L., Rizvi S. A. A., Jones W. (2009). CrystEngComm.

[cit54] Friščić T., Trask A. V., Jones W., Motherwell W. D. (2006). Angew Chem. Int. Ed. Engl..

[cit55] Azzali A., d'Agostino S., Capacci M., Spinelli F., Ventura B., Grepioni F. (2022). CrystEngComm.

[cit56] Jain H., Sutradhar D., Roy S., Desiraju G. R. (2021). Angew Chem. Int. Ed. Engl..

[cit57] Zander M., Franke W. (1958). Chem. Ber./Recl..

[cit58] Mu Z., Shu L., Fuchs H., Mayor M., Chi L. (2008). J. Am. Chem. Soc..

[cit59] Quo Y., Karasawa N., Goddard W. A. (1991). Nature.

[cit60] André D., Dworkin A., Szwarc H., Céolin R., Agafonov V., Fabre C., Rassat A., Straver L., Bernier P., Zahab A. (1992). Mol. Phys..

[cit61] Giri A., Hopkins P. E. (2017). J. Phys. Chem. Lett..

[cit62] Matsuno T., Nakai Y., Sato S., Maniwa Y., Isobe H. (2018). Nat. Commun..

[cit63] Clark S. J., Segall M. D., Pickard C. J., Hasnip P. J., Probert M. J., Refson K., Payne M. C. (2005). Z. Kristallogr. Cryst. Mater..

[cit64] Perdew J. P., Burke K., Ernzerhof M. (1996). Phys. Rev. Lett..

[cit65] Ehrlich S., Moellmann J., Reckien W., Bredow T., Grimme S. (2011). ChemPhysChem.

[cit66] Lisac K., Nemec V., Topić F., Arhangelskis M., Hindle P., Tran R., Huskić I., Morris A. J., Friščić T., Cinčić D. (2018). Cryst. Growth Des..

[cit67] Abe A., Goushi K., Mamada M., Adachi C. (2023). Adv. Mater..

[cit68] Zhan G., Liu Z., Bian Z., Huang C. (2019). Front. Chem..

[cit69] Yang J., Zhen X., Wang B., Gao X., Ren Z., Wang J., Xie Y., Li J., Peng Q., Pu K., Li Z. (2018). Nat. Commun..

[cit70] Su Y., Phua S. Z. F., Li Y., Zhou X., Jana D., Liu G., Lim W. Q., Ong W. K., Yang C., Zhao Y. (2018). Sci. Adv..

[cit71] Zhao W. J., He Z. K., Tang B. Z. (2020). Nat. Rev. Mater..

[cit72] Kenry, Chen C., Liu B. (2019). Nat. Commun..

[cit73] Hamzehpoor E., Ruchlin C., Tao Y., Liu C. H., Titi H. M., Perepichka D. F. (2023). Nat. Chem..

[cit74] An Z., Zheng C., Tao Y., Chen R., Shi H., Chen T., Wang Z., Li H., Deng R., Liu X., Huang W. (2015). Nat. Mater..

[cit75] Yang J., Zhen X., Wang B., Gao X., Ren Z., Wang J., Xie Y., Li J., Peng Q., Pu K., Li Z. (2018). Nat. Commun..

[cit76] Hamzehpoor E., Perepichka D. F. (2020). Angew. Chem., Int. Ed..

[cit77] Wang H., Hu R. X., Pang X., Gao H. Y., Jin W. J. (2014). CrystEngComm.

[cit78] Wei Y. C., Wang S. F., Hu Y., Liao L. S., Chen D. G., Chang K. H., Wang C. W., Liu S. H., Chan W. H., Liao J. L., Hung W. Y., Wang T. H., Chen P. T., Hsu H. F., Chi Y., Chou P. T. (2020). Nat. Photonics.

[cit79] Huang Y., Wang Z., Chen Z., Zhang Q. (2019). Angew Chem. Int. Ed. Engl..

[cit80] Sato K., Katoh R. (2019). Chem. Phys. Lett..

[cit81] Hestand N. J., Spano F. C. (2018). Chem. Rev..

