# Supplementary material for: Halogen bonding with carbon: directional assembly of non-derivatised aromatic carbon systems into robust supramolecular ladder architectures
Source: Chem Sci. 2023 Oct 24;14(45):13031–41. doi: 10.1039/d3sc04191c (PMC10664517; doi:10.1039/d3sc04191c)
Supplement: SC-014-D3SC04191C-s002 [file SC-014-D3SC04191C-s002.pdf]

Electronic Supplementary Material (ESI) for Chemical Science.
This journal is © The Royal Society of Chemistry 2023

Halogen bonding with carbon: directional assembly of non-derivatised aromatic carbon systems into robust supramolecular ladder architectures

Jogirdas Vainauskas[a,b], Tristan H. Borchers[a,b], Mihails Arhangelskis[c], Laura J. McCormick McPherson[d], Toni S. Spilfogel[a], Ehsan Hamzehpoor[b], Filip Topić[b], Simon J. Coles[d], Dmytro F. Perepichka[b], Christopher J. Barrett[b], Tomislav Friščić[a,b,*]

[a.] School of Chemistry, University of Birmingham, Edgbaston, Birmingham B15 2TT, UK. E-mail: t.friscic@bham.ac.uk

[b.] Department of Chemistry, McGill University, 801 Sherbrooke St. W. H3A 0B8 Montreal, Canada.

[c.] Faculty of Chemistry, University of Warsaw; 1 Pasteura Street, Warsaw 02-093, Poland.

[d.] EPSRC National Crystallography Service, School of Chemistry, University of Southampton, Highfield, Southampton, UK

	Page
1. Experimental/computational methods	2
2. Crystallographic data	5
3. Powder X-ray diffractograms	6
4. FTIR-ATR spectra	10
5. Differential scanning calorimetry	14
6. Thermogravimetric analysis	18
7. Analysis of Cambridge Structural Database (CSD)	22
8. Fluorescence and phosphorescence data	24
9. References	26

1. EXPERIMENTAL/COMPUTATIONAL METHODS

I. General information

All solvents for syntheses and crystal growth were of reagent grade and were used as received. 1,2,4,5-tetrafluoro-3,6-diiodobenzene (**14tfib**) was purchased from Oakwood Products, Inc, Anthracene (**anthra**) was purchased from Eastman Organic Chemicals, (**tet**) was purchased from TCI America, Inc., Pyrene (**pyr**) was purchased from Sigma Aldrich, Benzanthracene (**bant**) was purchased from TCI America, Inc., Perylene (**pery**) was purchased from TCI America, Inc., Coronene (**cor**) was purchased from TCI America, Inc and Buckminsterfullerene (**C_{60}**) was purchased from Sigma Aldrich.

II. Synthesis

Dicoronylene (**dicor**) was synthesized by a Scholl coupling of coronene in a melt of $AlCl_3$ and NaCl, following the procedure outlined by Zander et. al.,[1] and was purified by sublimation (See SI, Figure S7). Trans-2,3,5,6,2',3',5',6'-octafluoro-4,4'-diiodoazobenzene (ofiab) was synthesized in one step by treatment of 4-iodo-2,3,5,6-tetrafluoroaniline with N-chlorosuccinimide (NCS) and 1,8-diazabicyclo[5.4.0]undec-7-ene (DBU) in CH_2Cl_2 at -78°C.[2]

III. Mechanochemical ball milling

Mechanochemical ball milling was used to screen for cocrystal formation and composition, as well as for the preparation of cocrystals as bulk microcrystalline powders. Mechanochemical ball-milling was conducted using an FTS 1000 ball mill and analysis of the ball-milled reaction mixture was first conducted using PXRD. For cocrystal screening, the targeted cocrystal components (the arene and the XB donor) were milled in a range of stoichiometric compositions, permitting the discovery of new phases, as well as exploration of their composition. For ball-milling synthesis of bulk cocrystal materials, experiments were conducted on a 200 mg scale in which the solid components (in the appropriate stoichiometric ratios) were added into a 15 mL zirconia jar with a zirconia ball (~3 g), and milled at 30 Hz frequency. In the case of (**C_{60}**)(**14tfib**)$_2$, the zirconia jar was replaced with a 15 mL Teflon jar. Cocrystallization by liquid-assisted grinding (LAG) was conducted using nitromethane as the liquid additive (η=0.15 μL/mg)[3,4] in all cases, except for (**C_{60}**)(**14tfib**)$_2$ where toluene was used instead.

IV. Single crystal growth

(**bant**)$_2$(**14tfib**)$_5$, (**pery**)(**14tfib**)$_2$, (**cor**)(**14tfib**)$_2$, (**anthra**)(**ofiab**)$_2$, and (**pyr**)(**ofiab**)$_2$ single crystals were grown by recrystallization of ~10 mg of a sample obtained by LAG from DCM. (**tet**)(**14tfib**)$_2$ single crystals were grown by slow evaporation of a solution of ~1 mg of **tet** and ~ 5mg of **14tfib** in 1,2,4-trichlorobenzene. (**C_{60}**)(**14tfib**)$_2$ single crystals were grown by recrystallization of ~10 mg of mechanochemically prepared sample obtained by LAG from toluene. (**benz**)(**14tfib**) single crystals were grown by crystallization of ~10 mg of **14tfib** from **benz**, held at 4°C. (**pyr**)(**14tfib**)$_2$ single crystals were grown by dissolving ~10 mg of a sample obtained by LAG in DCM, heating the solution until boiling, immediately cooling in an ice bath for 10 seconds, followed by slow evaporation at room temperature. (**dicor**)(**14tfib**)$_3$ single crystals were grown by adding ~10 mg of **dicor** and ~100 mg of **14tfib** to a vial, heating the base of the vial to 110 °C in an oil bath, subsequently cooling at increments of 1°C every 2 hours until 106 °C and then holding at 106 °C overnight. The excess **14tfib** sublimed to the top of the vial, leaving behind red crystals at the base of the vial, which were used for single crystal x-ray diffraction.

V. Single crystal X-ray diffraction

Single crystal X-ray diffraction data (for all cocrystals except (**dicor**)(**14tfib**)$_3$ and (**bant**)$_2$(**14tfib**)$_5$) was collected on a Bruker D8 Venture dual-source diffractometer equipped with a PHOTON II detector and an Oxford Cryostream 800 cooling system, using mirror-monochromated CuK_α radiation (λ = 1.54184 Å) from a microfocus source. Data were collected in

a series of φ- and ω-scans. APEX3 software was used for data collection, integration and reduction.[5] Numerical absorption corrections were applied using SADABS-2016/2.[6]

Data for (**dicor**)(**14tfib**)$_3$ was collected on a Fluid Film Devices fixed kappa 3-circle diffractometer fitted with a Dectris PILATUS 2M detector on beamline I19-1 at Diamond Light Source. Synchrotron radiation of wavelength 0.6889(1) A, monochromated with a silicon double crystal monochromator. Data was collected at 100(2) K, and using the GDA software, but reprocessed using CrysAlisPro (1.171.41.114a (Rigaku OD, 2021)). Multi-scan absorption corrections were applied within CrysAlisPro using the ABSPACK scaling algorithm.

Data for (**bant**)$_2$(**14tfib**)$_5$ was collected on an Agilent SuperNova diffractometer using an Atlas detector. The data collection was driven, processed and an absorption correction was applied using CrysAlisPro.[7]

Structures were solved by dual-space iterative methods using SHELXT[8] and refined by full-matrix least-squares on *F*2 using all data with SHELXL[9] within the OLEX2[10] and/or SHELXLE8[11] environment. Some reflections were found to have been obscured by the beam stop and were omitted from the refinement. Extinction correction was applied for all structures. Hydrogen atoms were placed in calculated positions and treated as riding on the parent carbon atoms with isotropic displacement parameters 1.2 times larger than the respective parent atoms.

Crystal structure figures were generated using Mercury[12] and POV-Ray.[13]

VI. Powder X-ray diffraction (PXRD)

Powder X-ray diffraction experiments were performed on a Bruker D8 Advance diffractometer with CuK_α ($\lambda = 1.54184$ Å) radiation source operating at 40 mA and 40 kV, equipped with a Lynxeye XE linear position sensitive detector, a Bruker D2 Phaser diffractometer with CuK_α ($\lambda = 1.54184$ Å) radiation source operating at 10 mA and 30 kV, equipped with a Lynxeye linear position sensitive detector, in the 2θ range of 4/5−40°, or a PANalytical Empyrean diffractometer with CuK_α ($\lambda = 1.54184$ Å) radiation source operating at 30 mA and 40 kV.

VII. Fourier-transform infrared attenuated total reflectance (FTIR-ATR) spectroscopy

Fourier-transform infrared attenuated total reflectance (FTIR-ATR) measurements were performed on either a Bruker Alpha II instrument equipped with a single-reflection diamond crystal Platinum ATR unit, or a Perkin Elmer Spectrum Two equipped with a $LiTaO_3$ MIR detector.

VIII. Thermal analysis (TGA/DSC)

DSC measurements were performed using either a Mettler-Toledo DSC 1 Thermal Analyzer in hermetically-sealed aluminum pans (40 μL volume), heated in a stream of nitrogen (50 mL min−1) from 30 to 200 °C at a heating rate of 10 °C min−1, or a PerkinElmer DSC 8000 in an open alum pan from 30 to 200 °C at a heating rate of 10 °C min−1. Data collection and analysis were performed using the STARe Software 16.20 program package, or the Pyris Software package. Thermal Gravimetric Analysis (TGA) data was collected using a TGA5500 (TA instruments Ltd, Delaware, USA) or a PerkinElmer TGA 8000. All measurements, apart from (**bant**)$_2$(**14tfib**)$_5$ which was done under air, were done under nitrogen, in which the samples (*ca.* 5-10 mg) were placed in a Platinum pan and heated up to 400 or 600°C, using a constant heating ramp of 10 °C min^{-1}.

IX. Solid-state luminescence emission measurements

Solid-state luminescence spectra were collected using a Coherent OBIS 375 nm LX 50mW laser excitation source, modulated at differing laser powers 10-50 mW and an Ocean Optics QE65000 spectrometer. Lifetime measurements were performed using a Horiba Fluorolog.

X. Electrostatic potential map calculations

Electrostatic potential maps of molecules presented in this work were computed following molecular geometry optimization and population analysis using Gaussian16[14] employing the

PBEO hybrid functional theory,[15] basis set cc-PVTZ was used for halogen bond acceptors,[16] while def-TZVP was used for halogen bond donors.[17,18] The basis set for Iodine was sourced from basis set exchange.[19] Visualization of ESPs were computed in Vesta.[20]

XI. Periodic DFT calculations

Periodic DFT calculations were performed using the plane-wave DFT code CASTEP20.[21] The input files were generated from the CIF files of experimental crystal structures using the program cif2cell.[22] Crystal structures of the cocrystals and individual starting materials (CSD codes BENZEN, ANTCEN15, CORONE01, PERLEN03, PYRENE03, SOCTOT03, DOCDAC, YOFCUR, ZZZAVM02, TETCEN01, BEANTR) were geometry-optimized with respect to atom coordinates and unit cell parameters, taking into account the constraints imposed by the space-group symmetry. The calculations were performed with PBE[23] functional combined with Grimme D3 dispersion correction.[24] The plane-wave basis set was truncated at 800 eV cutoff, and ultra-soft built-in pseudopotentials were used to attenuate the Coulomb potentials in the atomic core regions. The 1st electronic Brillouin zone was sampled with $2\pi \times 0.05$ Å^{-1} k-point spacing. The convergence criteria were set with respect to total energy (2×10^{-5} eV/atom), maximum atomic force (0.05 eV Å^{-1}), maximum atom displacement (0.001 Å) and maximum component of the stress tensor (0.05 GPa). The energies associated with individual halogen bonds (E_{XB}) were obtained by subtracting the total energies of individual component molecules from the total energy of an individual halogen-bonded donor-acceptor dimer unit found in the cocrystal structure. The halogen-bonded dimer unit, or individual component molecules, were placed in a $30 \times 30 \times 30$ Å^3 simulation box, using the molecular geometries extracted from the optimized crystal structures. Plane-wave DFT single point energy calculations were subsequently performed using the same parameters as for the geometry optimizations to generate the associated energy values.

XII. Search of the Cambridge Structural Database

The search was conducted using the Conquest software (CCDC version 2020.3),[25] targeting intermolecular distances (in Å) involving the iodine atom of a C-I fragment and a carbon atom of the 5-, 6- or 7- membered ring, a C=C or a C≡C fragment. The C-I···C_π angle was also recorded and constrained to the 90-180° range. To maximize search scope, the type of all covalent carbon-carbon bonds in the 5-, 6- and 7-membered rings, as well as C=C or a C≡C fragments was set to "any", in order to avoid potentially mis-assigned bond types. The aromaticity of the carbon atoms was then accounted for by restraining each carbon atom to be covalently bonded with no more than three partners in case of C=C bond fragments and ring systems, and no more than two for C≡C bond fragments. To avoid potential overlap between search results from searches targeting aromatic rings and the C=C bond fragments, the CSD hits from the former search were excluded from the search involving the double bond fragment. As XBs are expected to be shorter than the sum of the van der Waals radii of interacting atoms, the search was limited to include all I···C distances up to 4.68 Å, which is 1 Å longer than the anticipated sum of van der Waals radii for carbon (1.7 Å) and iodine (1.98 Å) atoms.[26] Plots of the distribution of C-I···C_π contact lengths and angles for alkyne (C≡C) and 6-membered ring acceptors are given in Figure 4, while the values for alkene (C=C), 5- and 7-membered ring acceptors are given in the SI.

2. Crystallographic Data

Supplementary Table S1. Crystallographic data for all the crystal structures reported in this work.

**Identification code**	**(anthra)(14tfib)$_2$**	**(anthra)(ofiab)$_2$**	**(benz)(14tfib)**	**(bant)$_2$(14tfib)$_5$**	**(C_{60})(14tfib)$_2$**	**(cor)(14tfib)$_2$**	**(dicor)(14tfib)$_3$**	**(pery)(14tfib)$_2$**	**(pyr)(ofiab)$_2$**	**(pyr)(14tfib)$_2$**	**(tet)(14tfib)**
**Empirical formula**	$C_{26}H_{10}F_8I_4$	$C_{19}H_5F_8I_2N_2$	$C_{12}H_6F_4I_{1.99}$	$C_{33}H_{12}F_{10}I_5$	$C_{72}F_8I_4$	$C_{36}H_{12}F_8I_4$	$C_{66}H_{20}F_{12}I_6$	$C_{32}H_{12}F_8I_4$	$C_{20}H_5F_8I_2N_2$	$C_{14}H_5F_4I_2$	$C_{24}H_{12}F_4I_2$
**Formula weight**	981.94	667.05	478.70	1232.93	1524.32	1104.06	1802.22	1056.02	679.06	502.98	630.14
**Temperature/K**	273(2)	298(2)	180(2)	291.9(4)	298(2)	183(2)	100(2)	298(2)	298(2)	273(2)	298(2)
**Crystal system**	triclinic	triclinic	triclinic	triclinic	orthorhombic	triclinic	triclinic	triclinic	triclinic	triclinic	monoclinic
**Space group**	P-1	P-1	P-1	P-1	Cmce	P-1	P-1	P-1	P-1	P-1	P2$_1$/c
**a/Å**	5.9679(8)	5.9146(8)	5.6444(7)	6.1683(5)	24.9493(4)	5.7028(6)	5.1812(2)	5.8113(2)	6.04030(10)	6.1992(3)	19.1824(19)
**b/Å**	9.6419(13)	9.8631(14)	5.6487(7)	15.3618(13)	17.2771(3)	10.2278(12)	15.4603(5)	10.2259(4)	12.6794(3)	9.2122(5)	5.9202(7)
**c/Å**	12.2058(16)	17.9820(18)	10.5675(13)	18.5460(13)	10.18670(10)	14.3969(15)	17.4682(6)	13.3298(4)	13.4013(4)	12.9134(7)	8.9814(9)
**α/°**	73.039(5)	94.026(5)	90.233(7)	94.961(6)	90	104.396(6)	72.718(3)	103.6520(10)	88.433(2)	107.150(2)	90
**β/°**	89.800(5)	97.548(5)	90.118(7)	93.811(6)	90	97.493(5)	87.214(3)	92.767(2)	79.590(2)	94.642(2)	90.617(6)
**γ/°**	81.559(5)	105.520(6)	100.763(8)	97.532(7)	90	95.668(6)	81.882(3)	96.252(2)	84.594(2)	97.251(2)	90
**Volume/Å^3**	663.94(15)	995.9(2)	331.00(7)	1730.3(2)	4390.99(11)	798.86(15)	1322.66(8)	762.93(5)	1004.93(4)	693.58(6)	1019.90(19)
**Z**	1	2	1	2	4	1	1	1	2	2	2
**ρ_{calc}g/cm^3**	2.456	2.224	2.402	2.366	2.306	2.295	2.263	2.298	2.244	2.408	2.052
**μ/mm^{-1}**	37.522	25.594	37.423	36.001	23.071	31.298	3.346	32.725	25.381	35.945	24.621
**F(000)**	450.0	622.0	219.0	1130	2864.0	512.0	842.0	488.0	634.0	462.0	596.0
**Crystal size/mm^3**	0.352 × 0.145 × 0.055	0.330 × 0.150 × 0.075	0.310 × 0.300 × 0.170	0.17 × 0.12 × 0.06	0.334 × 0.151 × 0.093	0.29 × 0.21 × 0.06	0.27 × 0.01 × 0.01	0.400 × 0.200 × 0.130	0.31 × 0.16 × 0.08	0.315 × 0.175 × 0.090	0.16 × 0.152 × 0.043
**Radiation**	CuKα (λ = 1.54178)	CuKα (λ = 1.54178)	CuKα (λ = 1.54178)	Cu Kα (λ = 1.54184)	CuKα (λ = 1.54178)	CuKα (λ = 1.54178)	synchrotron (λ = 0.6889)	CuKα (λ = 1.54178)	CuKα (λ = 1.54178)	CuKα (λ = 1.54178)	CuKα (λ = 1.54178)
**2Θ range for data collection/°**	9.704 to 145.268	4.988 to 145.11	8.366 to 145.038	7.902 to 145.28	7.086 to 144.936	9.01 to 145.494	3.022 to 49.026	6.844 to 144.784	6.706 to 144.916	7.22 to 145.464	4.606 to 145.078
**Data completeness %**	97.7	98.8	98.7	97.3	99.8	98.9	98.2	98.8	99.0	98.9	99.9
**Index ranges**	-7 ≤ h ≤ 7, -11 ≤ k ≤ 11, -14 ≤ l ≤ 15	-7 ≤ h ≤ 7, -12 ≤ k ≤ 12, -22 ≤ l ≤ 22	-6 ≤ h ≤ 6, -6 ≤ k ≤ 6, -13 ≤ l ≤ 13	-7 ≤ h ≤ 7, -18 ≤ k ≤ 19, -22 ≤ l ≤ 19	-30 ≤ h ≤ 30, -21 ≤ k ≤ 14, -11 ≤ l ≤ 12	-7 ≤ h ≤ 5, -12 ≤ k ≤ 12, -17 ≤ l ≤ 17	-6 ≤ h ≤ 6, -18 ≤ k ≤ 18, -21 ≤ l ≤ 21	-6 ≤ h ≤ 7, -12 ≤ k ≤ 12, -16 ≤ l ≤ 16	-7 ≤ h ≤ 7, -14 ≤ k ≤ 15, -16 ≤ l ≤ 16	-7 ≤ h ≤ 7, -11 ≤ k ≤ 11, -15 ≤ l ≤ 15	-23 ≤ h ≤ 23, -5 ≤ k ≤ 7, -11 ≤ l ≤ 11
**Reflections collected**	9449	14419	3945	14023	35985	10608	15312	12061	16872	13339	15463
**Independent reflections**	2574 [R_{int} = 0.0571, R_{sigma} = 0.0533]	3908 [R_{int} = 0.0711, R_{sigma} = 0.0645]	1273 [R_{int} = 0.0731, R_{sigma} = 0.0695]	6690 [R_{int} = 0.0497, R_{sigma} = 0.0642]	2236 [R_{int} = 0.0678, R_{sigma} = 0.0289]	3107 [R_{int} = 0.0494, R_{sigma} = 0.0496]	4751 [R_{int} = 0.0678, R_{sigma} = 0.0934]	2980 [R_{int} = 0.0667, R_{sigma} = 0.0589]	3937 [R_{int} = 0.0502, R_{sigma} = 0.0414]	2721 [R_{int} = 0.0519, R_{sigma} = 0.0410]	2020 [R_{int} = 0.0806, R_{sigma} = 0.0451]
**Data/restraints/parameters**	2574/117/173	3908/216/281	1273/37/83	6690/348/421	2236/171/194	3107/174/217	4751/412/452	2980/159/200	3937/234/290	2721/123/182	2020/73/137
**Goodness-of-fit on F^2**	1.036	1.073	1.086	1.047	1.094	1.085	0.917	1.100	1.090	1.131	1.077
**Final R indexes [I>=2σ (I)]**	R_1 = 0.0590, wR_2 = 0.1599	R_1 = 0.0827, wR_2 = 0.2207	R_1 = 0.0910, wR_2 = 0.2172	R_1 = 0.0750, wR_2 = 0.2011	R_1 = 0.0434, wR_2 = 0.1103	R_1 = 0.0697, wR_2 = 0.1745	R_1 = 0.0705, wR_2 = 0.2266	R_1 = 0.0857, wR_2 = 0.2115	R_1 = 0.0492, wR_2 = 0.1466	R_1 = 0.0482, wR_2 = 0.1146	R_1 = 0.0464, wR_2 = 0.0995
**Final R indexes [all data]**	R_1 = 0.0631, wR_2 = 0.1708	R_1 = 0.1068, wR_2 = 0.2396	R_1 = 0.0959, wR_2 = 0.2304	R_1 = 0.1268, wR_2 = 0.2656	R_1 = 0.0554, wR_2 = 0.1282	R_1 = 0.0889, wR_2 = 0.1923	R_1 = 0.0845, wR_2 = 0.2438	R_1 = 0.0950, wR_2 = 0.2304	R_1 = 0.0656, wR_2 = 0.1699	R_1 = 0.0550, wR_2 = 0.1207	R_1 = 0.0614, wR_2 = 0.1107
**Largest diff. peak/hole / e Å^{-3}**	1.27/-1.67	1.09/-0.92	4.27/-2.40	2.13/-1.64	1.10/-0.75	3.31/-2.27	1.46/-1.16	2.19/-3.18	1.23/-1.31	1.58/-2.43	0.73/-1.28

Supplementary Table S2. Short contact distances between iodine and carbon (<3.68 Å), corresponding interaction angles, and contact distances between iodine and the carbon-carbon centroid with relative standard deviations for herein reported cocrystals.

Identification code	$d_{I\cdots C}$ (Å)	∠C-I···C (°)	$d_{I\cdots C\text{-}C\ (centroid)}$ (Å)
(**benz**)(**14tfib**)	3.48(2), 3.51(2)	165.2(4), 165.7(4)	3.42(1)
(**anthra**)(**14tfib**)$_2$	3.427(9), 3.445(8), 3.583(7)	173.2(2), 157.2(2), 169.4(2)	3.366(5)
(**tet**)(**14tfib**)	3.410(6), 3.509(7)	162.5(2), 173.6(2)	3.387(5)
(**bant**)$_2$(**14tfib**)$_5$	3.50(2), 3.64(2), 3.50(1), 3.46(1), 3.51(3), 3.39(2), 3.48(2), 3.40(2), 3.55(1)	174.2(5), 158.5(5), 170.5(4), 162.9(4), 167.4(6), 168.2(5), 160.6(5), 167.6(5), 172.1(4)	3.49(1), 3.409(8), 3.39(1), 3.37(1)
(**pyr**)(**14tfib**)$_2$	3.515(8), 3.56(1), 3.50(1), 3.50(1)	160.3(3), 165.6(3), 159.3(3), 171.1(3)	3.430(7), 3.467(6)
(**pery**)(**14tfib**)$_2$	3.509(9), 3.430(8), 3.482(9), 3.51(1)	156.0(3), 175.2(3), 168.4(3), 166.4(3)	3.394(6), 3.433(6)
(**cor**)(**14tfib**)$_2$	3.45(1), 3.46(2), 3.46(1), 3.51(2)	164.0(4), 172.4(4), 163.3(4), 165.5(4)	3.384(8), 3.415(8)
(**dicor**)(**14tfib**)$_3$	3.440(8), 3.424(7), 3.368(8), 3.38(1), 3.560(9)	176.7(3), 163.0(3), 163.0(3), 178.4(4), 158.5(4)	3.473(9), 3.400(7), 3.579(8), 3.327(6)
(**C$_{60}$**)(**14tfib**)$_2$	3.54(1)	162.8(2)	3.545(6)
(**anthra**)(**ofiab**)$_2$	3.62(2), 3.43(1), 3.55(2), 3.63(2)	158.1(5), 178.3(4), 172.0(5), 158.7(5)	3.518(9), 3.457(8)
(**pyr**)(**ofiab**)$_2$	3.476(7), 3.394(7), 3.586(7), 3.448(6)	171.2(2), 165.6(2), 166.1(2), 170.6(2)	3.369(5), 3.446(5)

2. POWDER X-RAY DIFFRACTOGRAMS

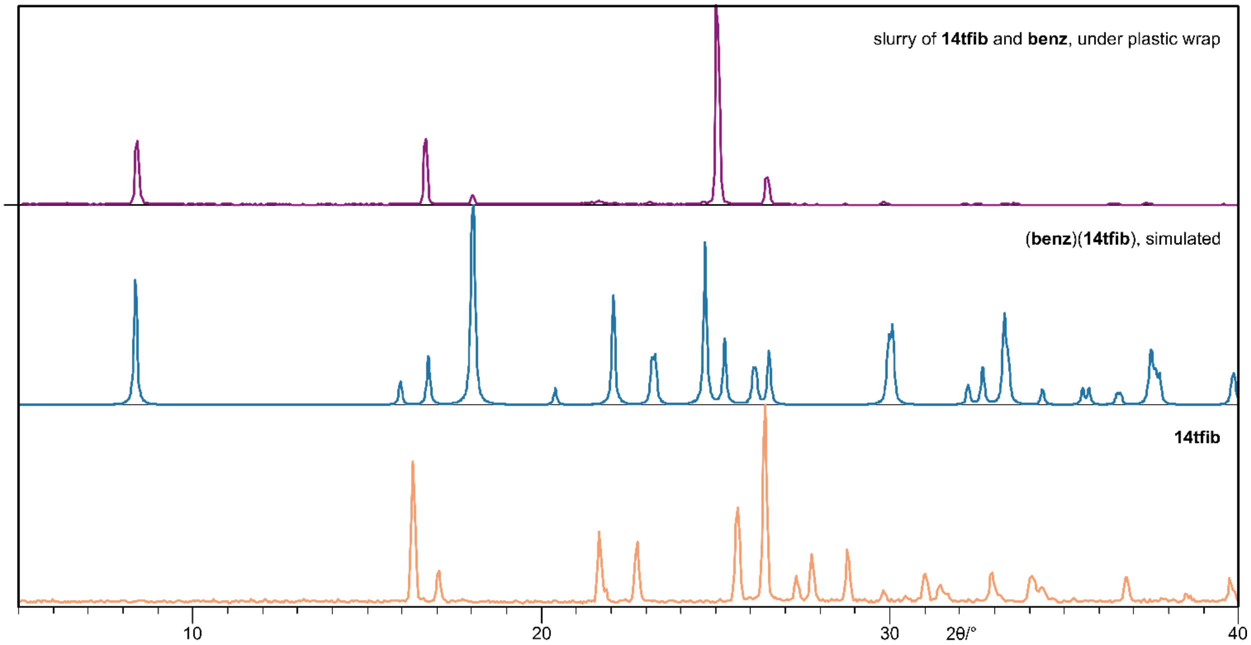

Supplementary Figure S1. PXRD diffractograms for slurry experiment of **14tfib** and **benz**, including simulated pattern for the cocrystal and **14tfib** starting material.

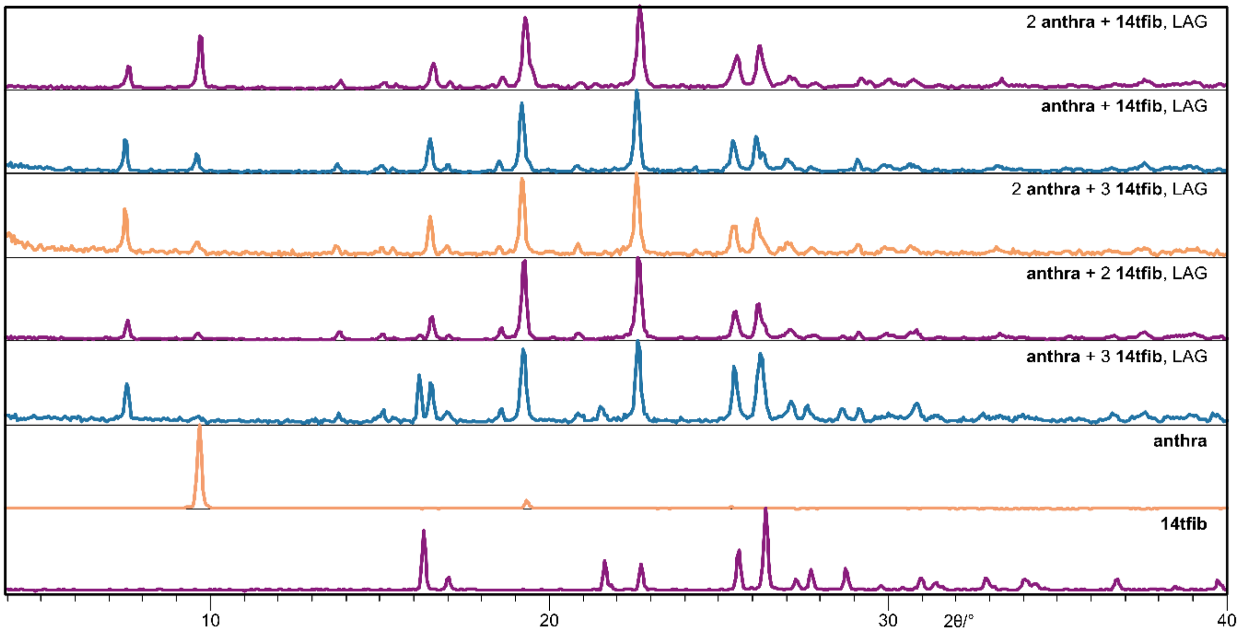

Supplementary Figure S2. Selected PXRD patterns for mechanochemical neat milling and LAG screening for cocrystal formation involving **anthra** and **14tfib**, based on varying **anthra**:**14tfib** stoichiometries.

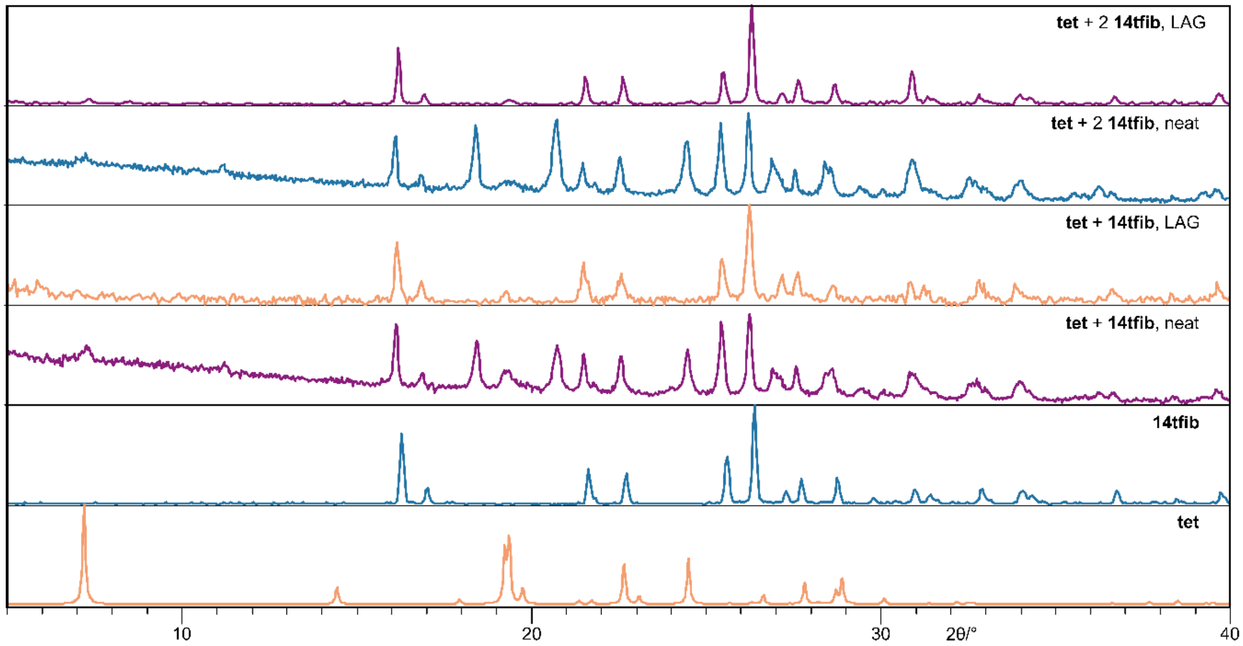

Supplementary Figure S3. Selected PXRD patterns for mechanochemical neat milling and LAG screening for cocrystal formation involving **tet** and **14tfib**, based on varying **tet**:**14tfib** stoichiometries.

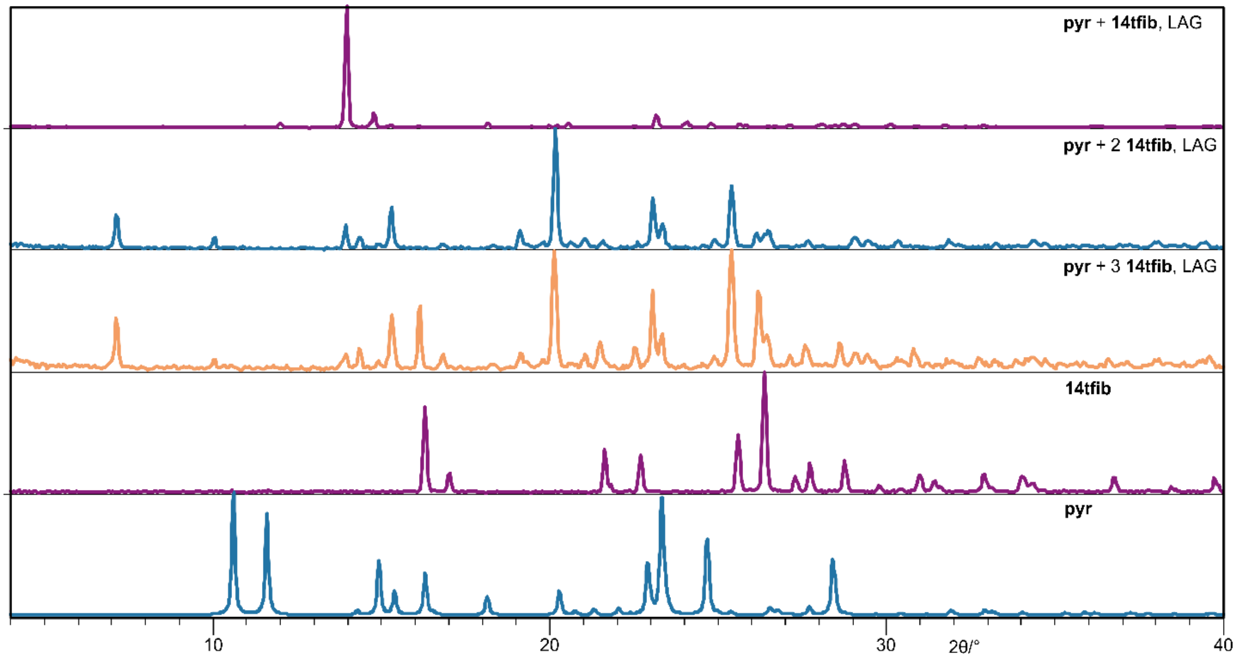

Supplementary Figure S4. Selected PXRD patterns for mechanochemical LAG screening for cocrystal formation involving **pyr** and **14tfib**, based on varying **pyr**:**14tfib** stoichiometries.

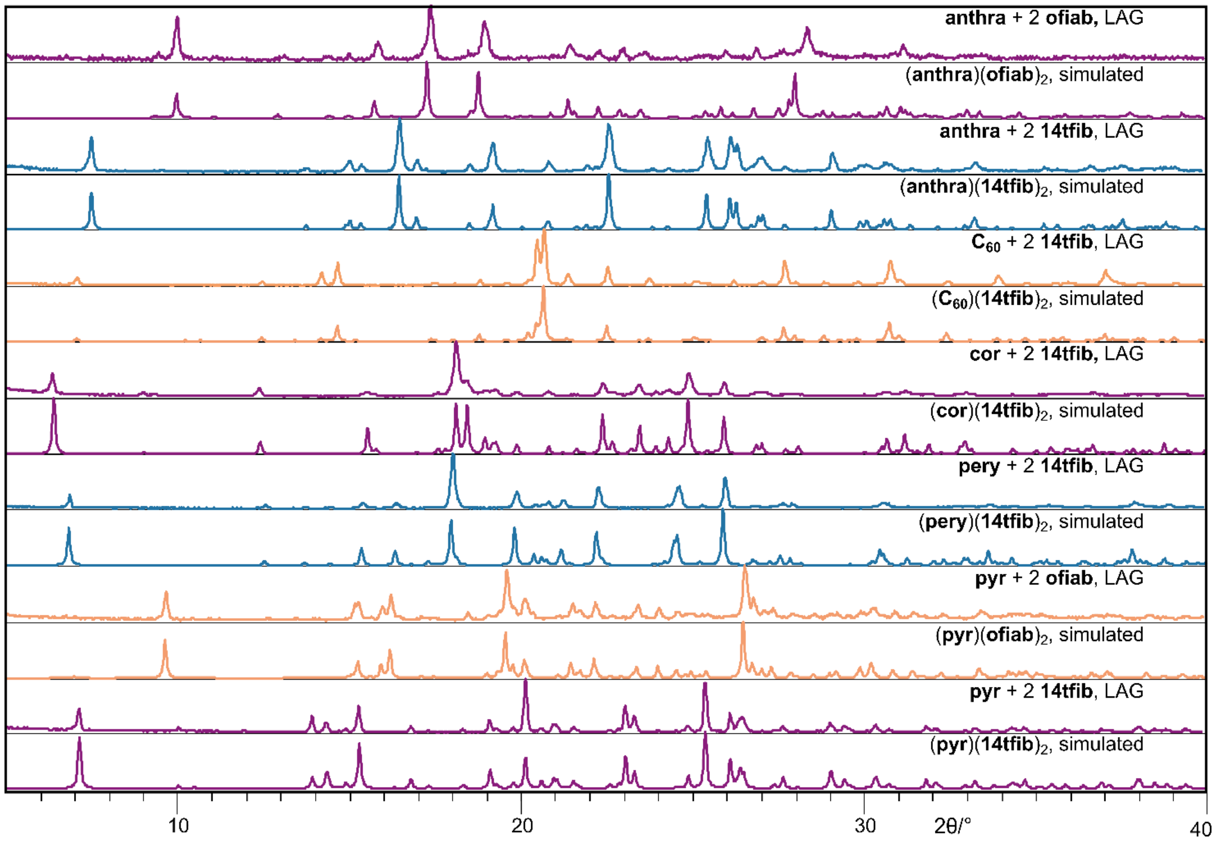

Supplementary Figure S5. Overview of PXRD patterns for mechanochemically prepared cocrystals, compared to patterns simulated based on the respective single crystal structures.

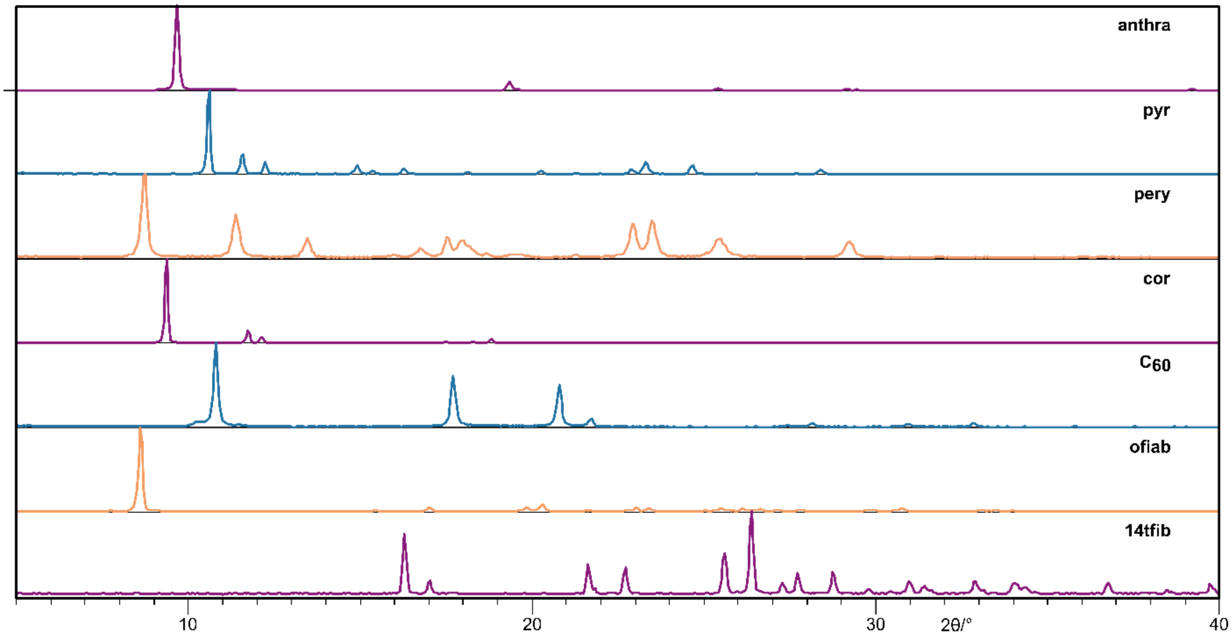

Supplementary Figure S6. PXRD diffractograms for solid starting materials used in mechanochemical ball milling experiments.

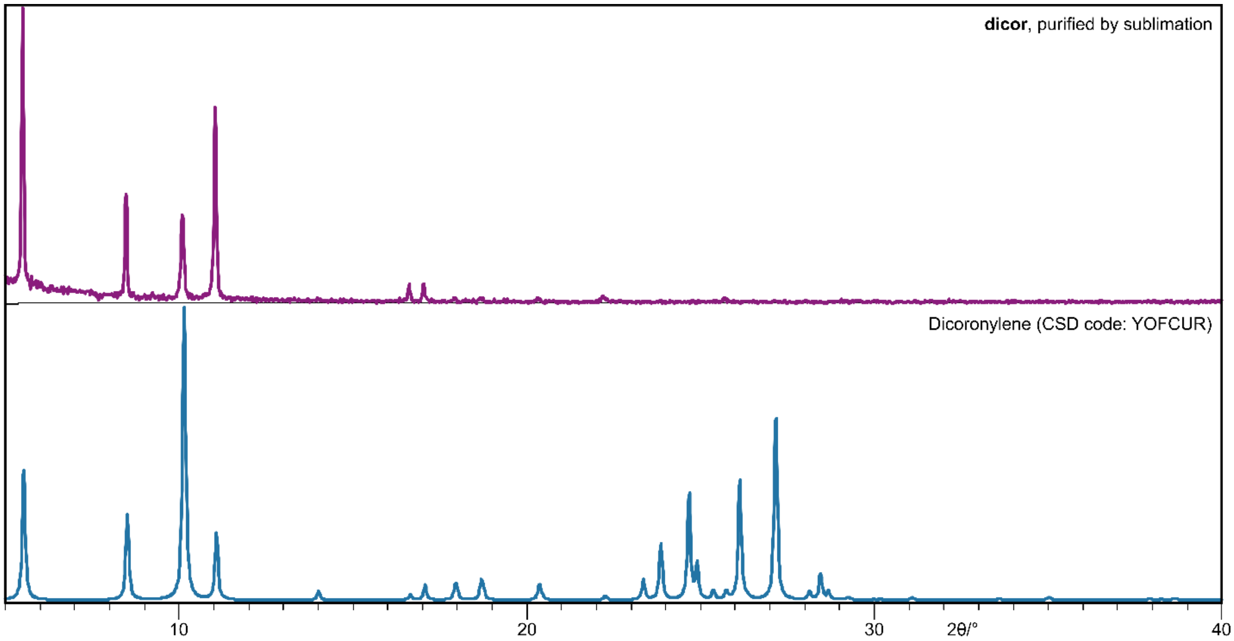

Supplementary Figure S7. Comparison of the PXRD patterns for synthesized **dicor** and simulated for the previously reported crystal structure of **dicor** (CSD code YOFCUR).

3. FTIR-ATR SPECTRA

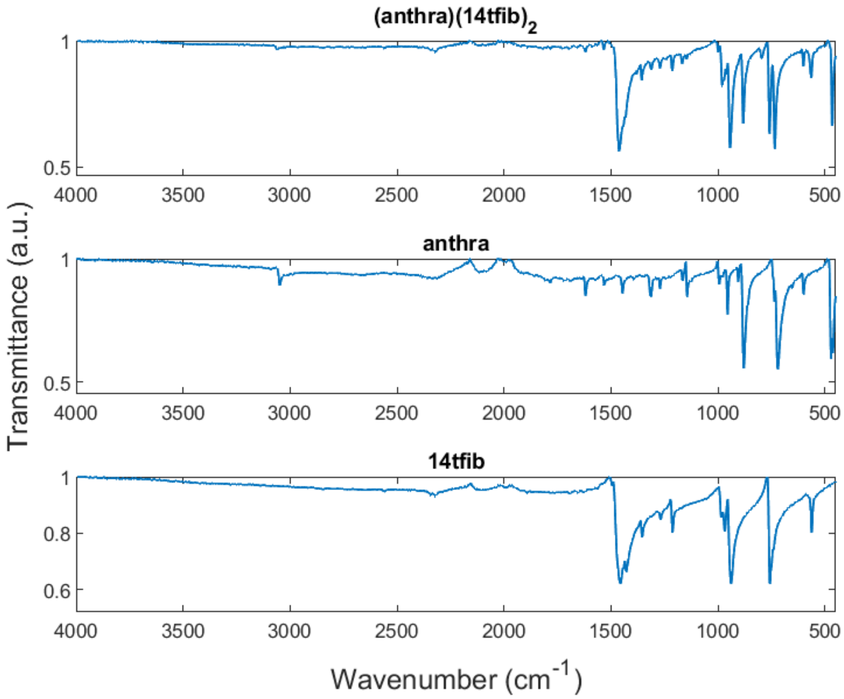

Supplementary Figure S8. Comparison of FTIR-ATR spectra for the cocrystal (**anthra**)(**14tfib**)$_2$, **anthra** and **14tfib**.

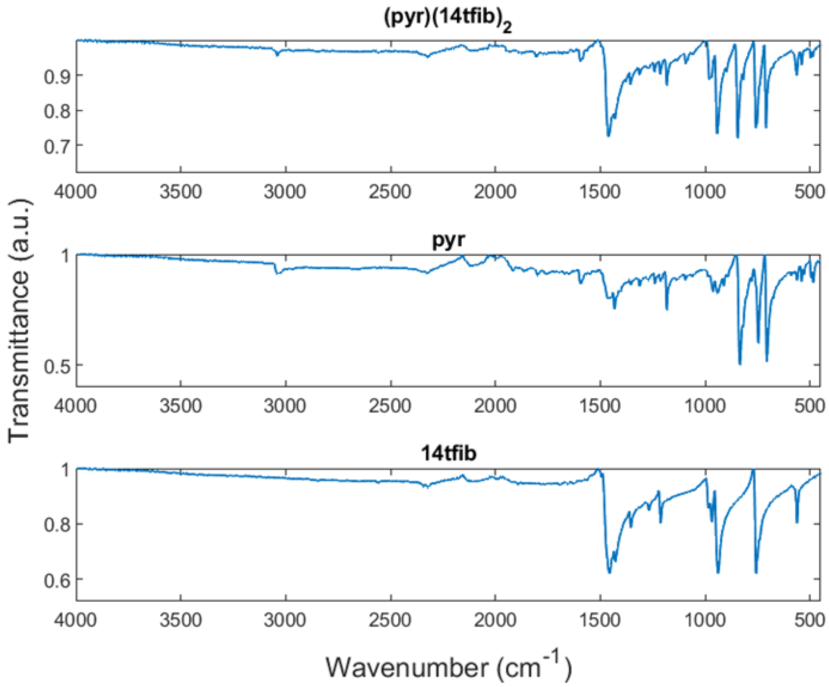

Supplementary Figure S9. Comparison of FTIR-ATR spectra for the cocrystal (**pyr**)(**14tfib**)$_2$, **pyr** and **14tfib**.

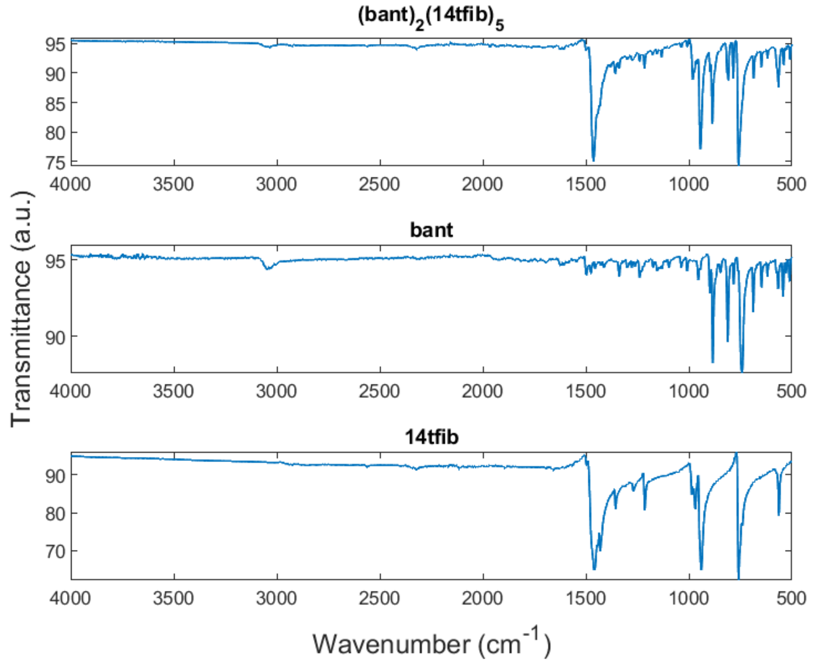

Supplementary Figure S10. Comparison of FTIR-ATR spectra for the cocrystal (**bant**)$_2$(**14tfib**)$_5$, **bant** and **14tfib**.

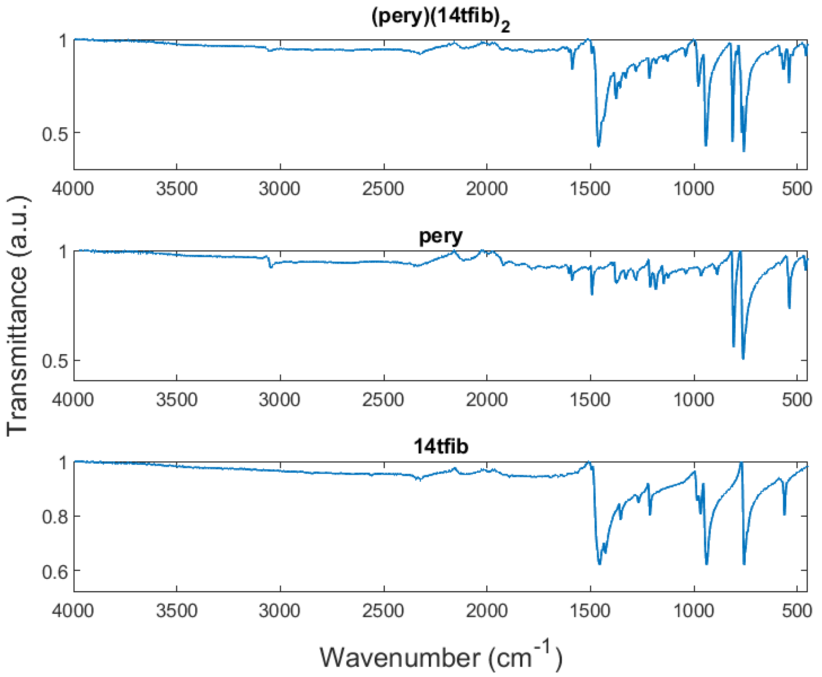

Supplementary Figure S11. Comparison of FTIR-ATR spectra for the cocrystal (**pery**)(**14tfib**)$_2$, **pery** and **14tfib**.

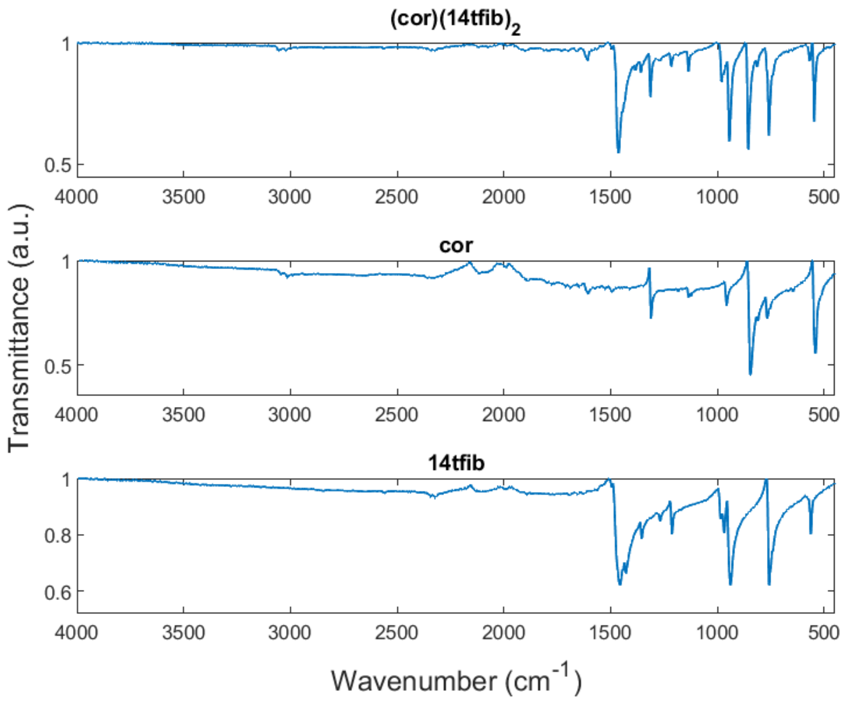

Supplementary Figure S12. Comparison of FTIR-ATR spectra for the cocrystal (**cor**)(**14tfib**)$_2$, **cor** and **14tfib**.

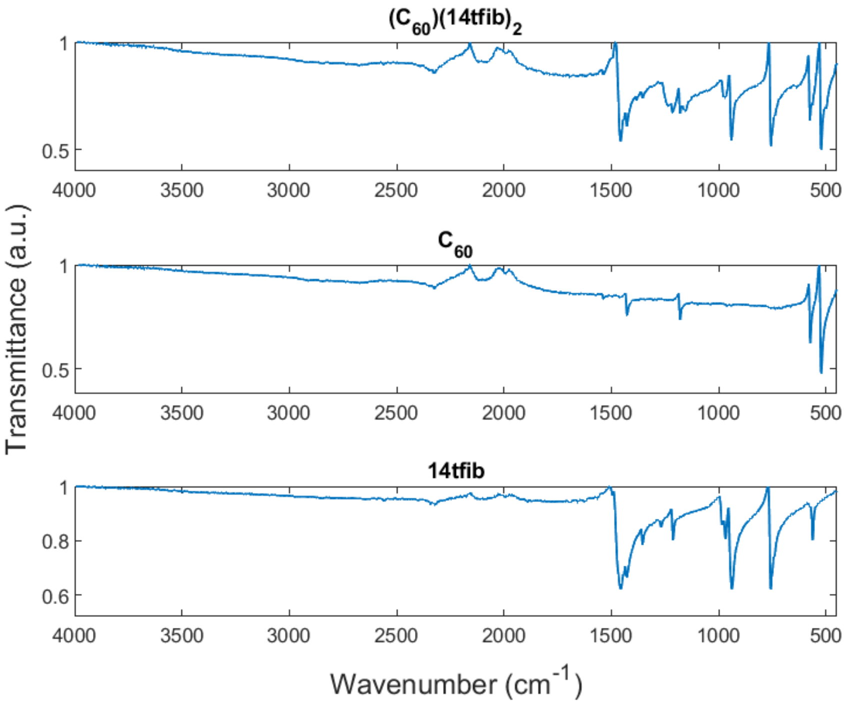

Supplementary Figure S13. Comparison of FTIR-ATR spectra for the cocrystal (**C$_{60}$**)(**14tfib**)$_2$, **C$_{60}$** and **14tfib**.

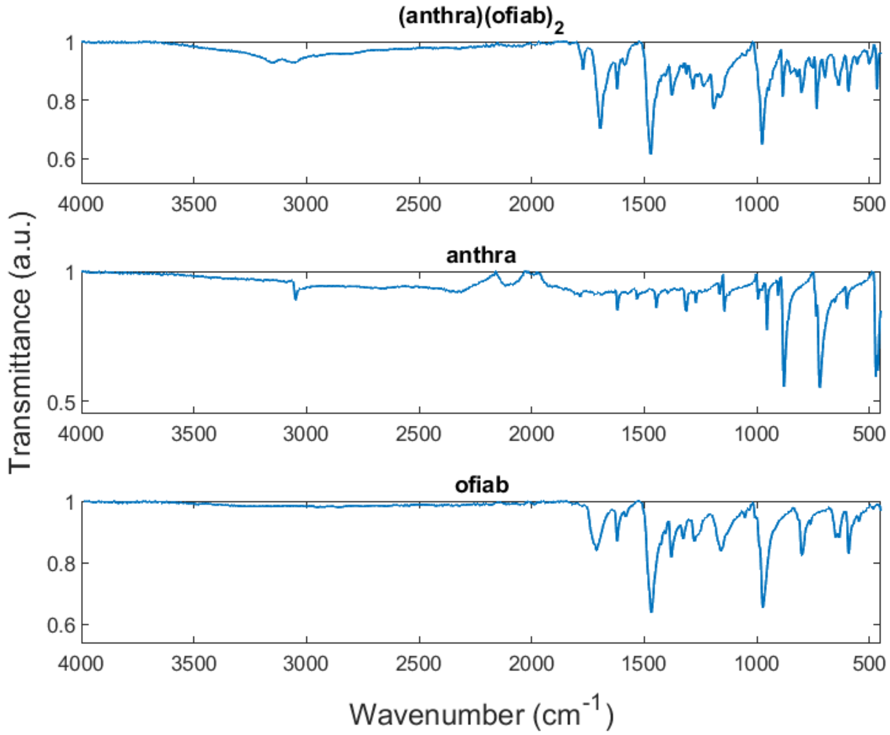

Supplementary Figure S14. Comparison of FTIR-ATR spectra for the cocrystal (**anthra**)(**ofiab**)$_2$, **anthra** and **ofiab**.

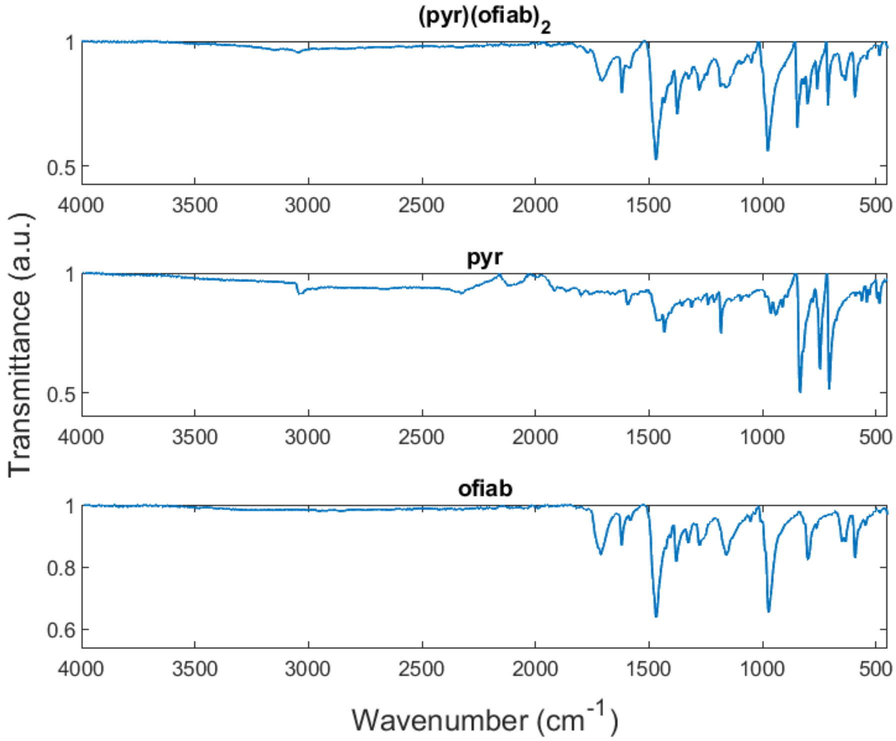

Supplementary Figure S15. Comparison of FTIR-ATR spectra for the cocrystal (**pyr**)(**ofiab**)$_2$, **pyr** and **ofiab**.

4. Differential Scanning Calorimetry

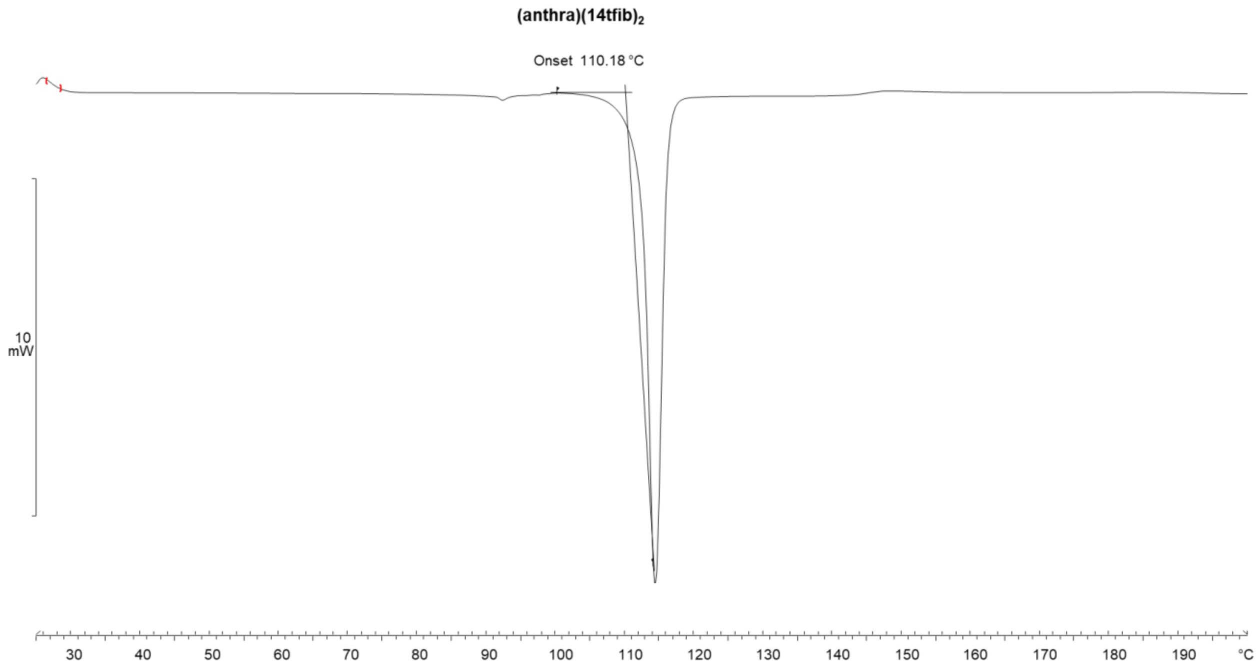

Supplementary Figure S16. DSC thermogram for (**anthra**)(**14tfib**)$_2$ (25-200 °C, heating rate 10 °C min^{-1} in N_2 atmosphere) (reported melting points for **anthra** and **14tfib** are 216°C and 106°C, respectively).[27,28]

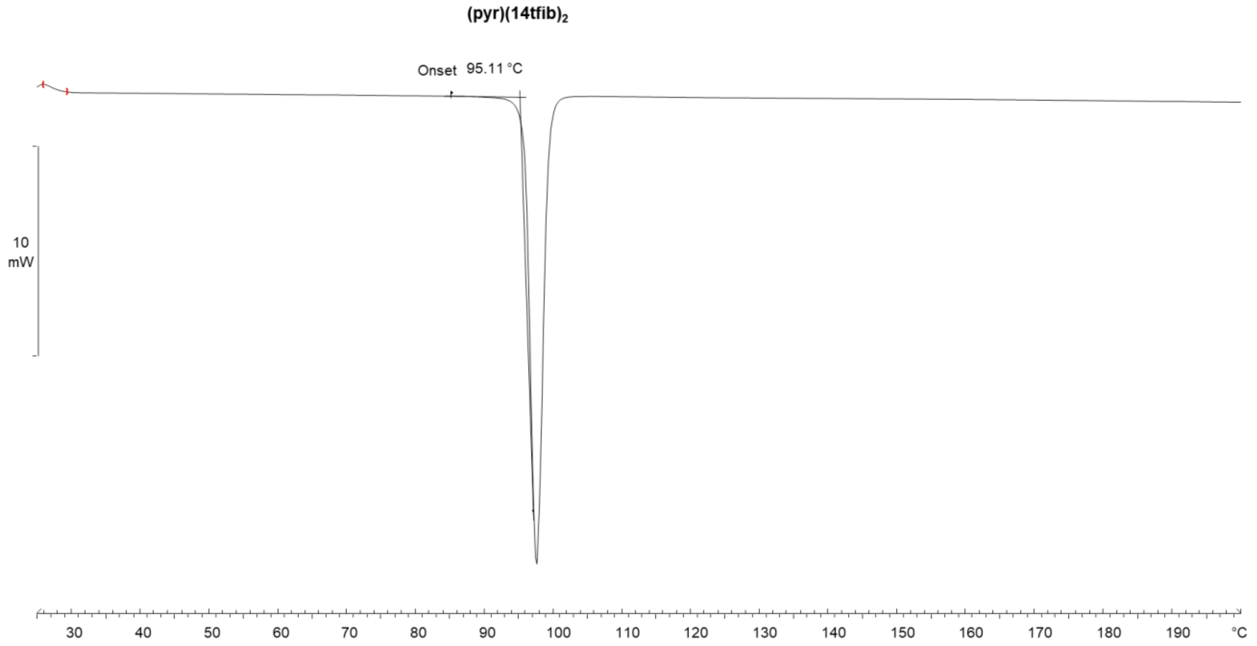

Supplementary Figure S17. DSC thermogram for (**pyr**)(**14tfib**)$_2$ (25-200 °C, heating rate 10 °C min^{-1} in N_2 atmosphere) (reported melting points for **pyr** and **14tfib** are 150°C and 106°C, respectively).[27,28]

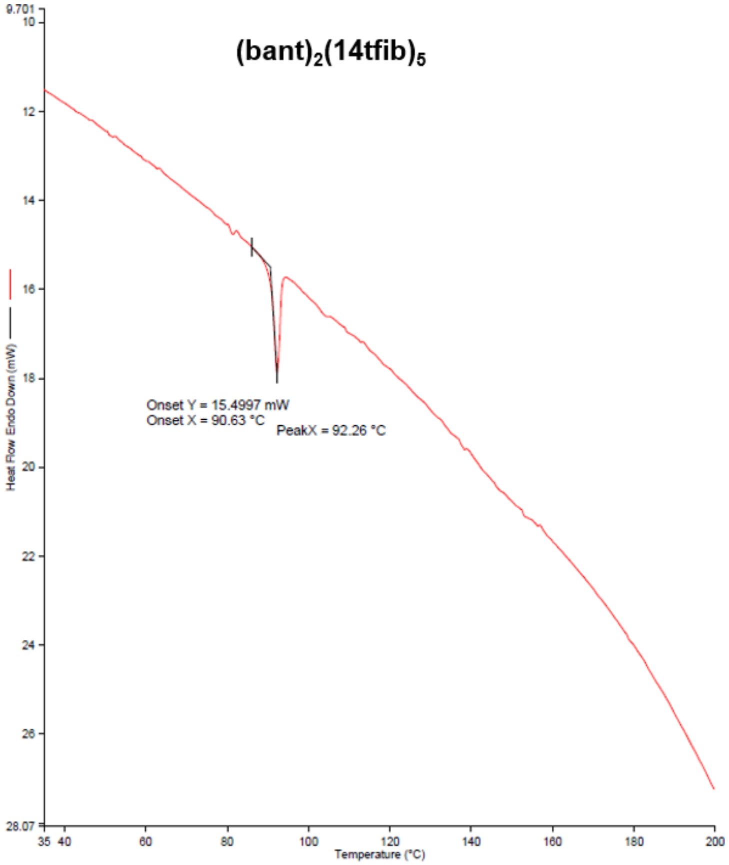

Supplementary Figure S18. DSC thermogram for (**bant**)$_2$(**14tfib**)$_5$ (30-200 °C, heating rate 10 °C min^{-1} in N_2 atmosphere) (reported melting points for **bant** and **14tfib** are 158°C and 106°C, respectively).[27,28]

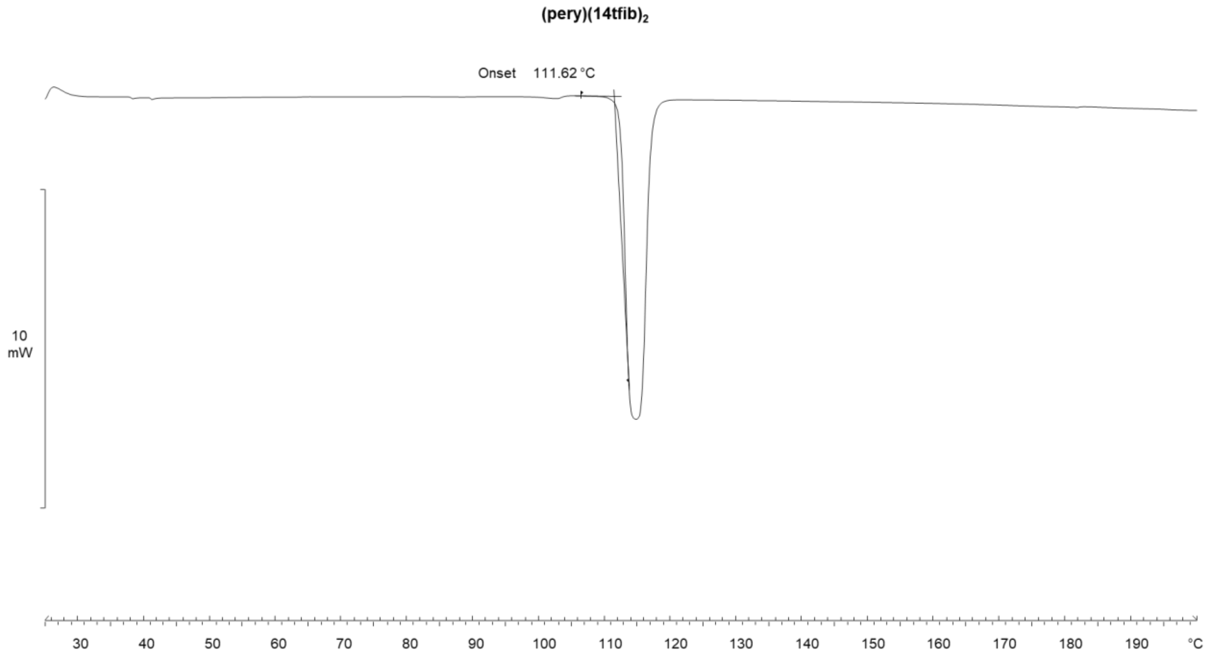

Supplementary Figure S19. DSC thermogram for (**pery**)(**14tfib**)$_2$ (25-200 °C, heating rate 10 °C min^{-1} in N_2 atmosphere) (reported melting points for **pery** and **14tfib** are 276°C and 106°C, respectively).[27,28]

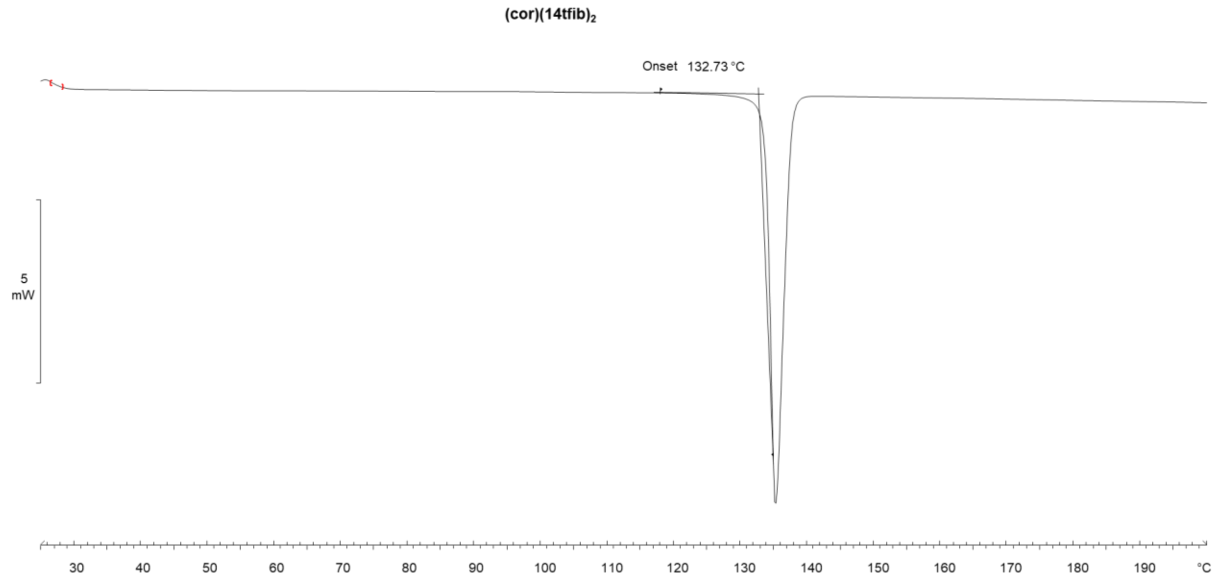

Supplementary Figure S20. DSC thermogram for (**cor**)(**14tfib**)$_2$ (25-200 °C, heating rate 10 °C min^{-1} in N_2 atmosphere) (reported melting points for **cor** and **14tfib** are 437°C and 106°C, respectively). [27,28]

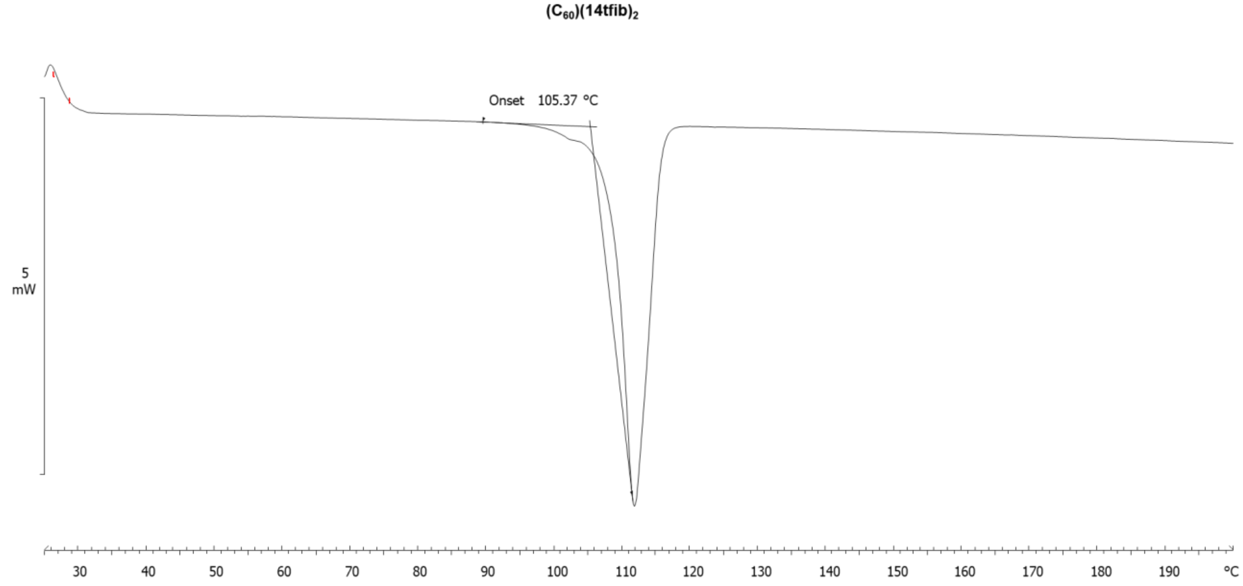

Supplementary Figure S21. DSC thermogram for (**C_{60}**)(**14tfib**)$_2$ (25-200 °C, heating rate 10 °C min^{-1} in N_2 atmosphere) (reported melting points for **C_{60}** and **14tfib** are >280°C and 106°C, respectively). [27,28]

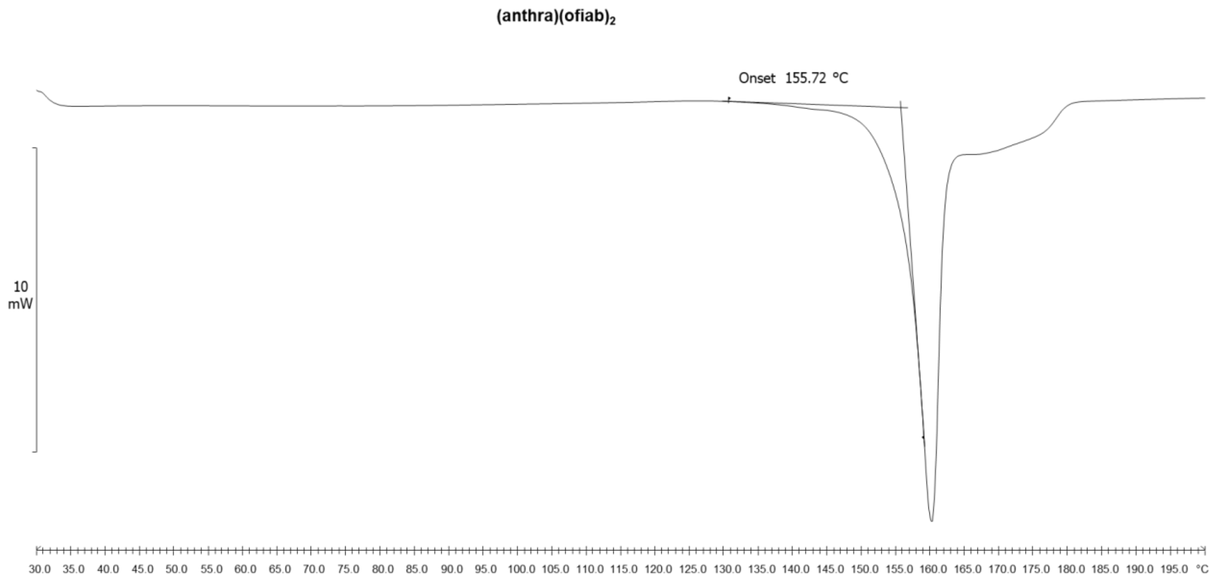

Supplementary Figure S22. DSC thermogram for (**anthra**)(**ofiab**)$_2$ (30-200 °C, heating rate 10 °C min^{-1} in N_2 atmosphere) (reported melting points for **anthra** and **14tfib** are 216°C and 198°C, respectively). [27,29]

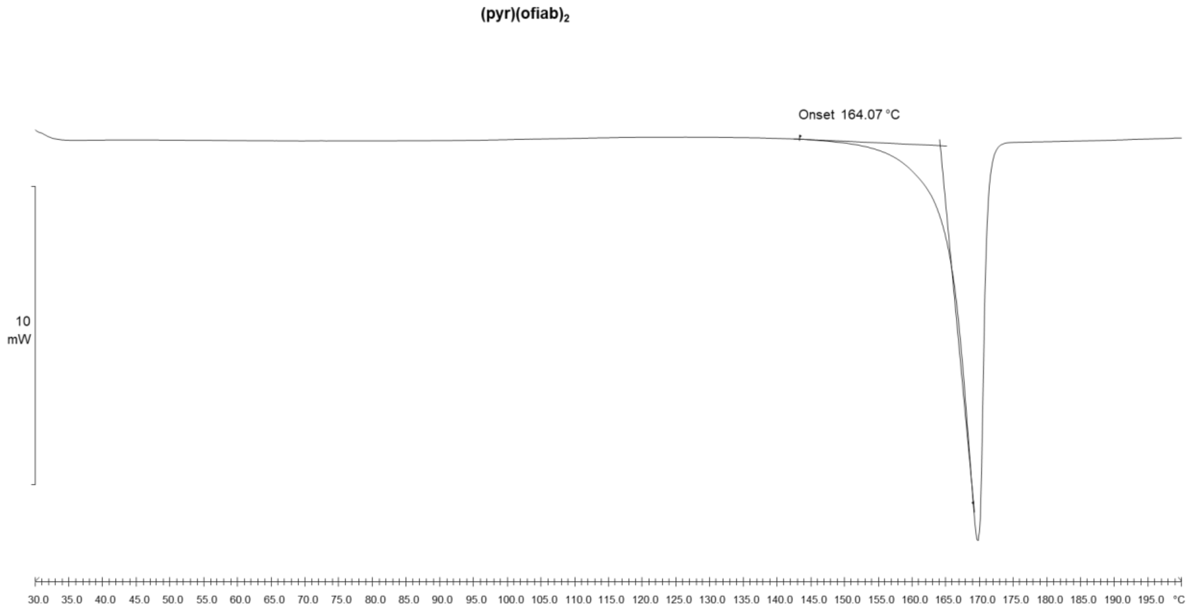

Supplementary Figure S23. DSC thermogram for (**pyr**)(**ofiab**)$_2$ (30-200 °C, heating rate 10 °C min^{-1} in N_2 atmosphere) (reported melting points for **pyr** and **ofiab** are 150°C and 198°C, respectively). [27,29]

5. THERMOGRAVIMETRIC ANALYSIS

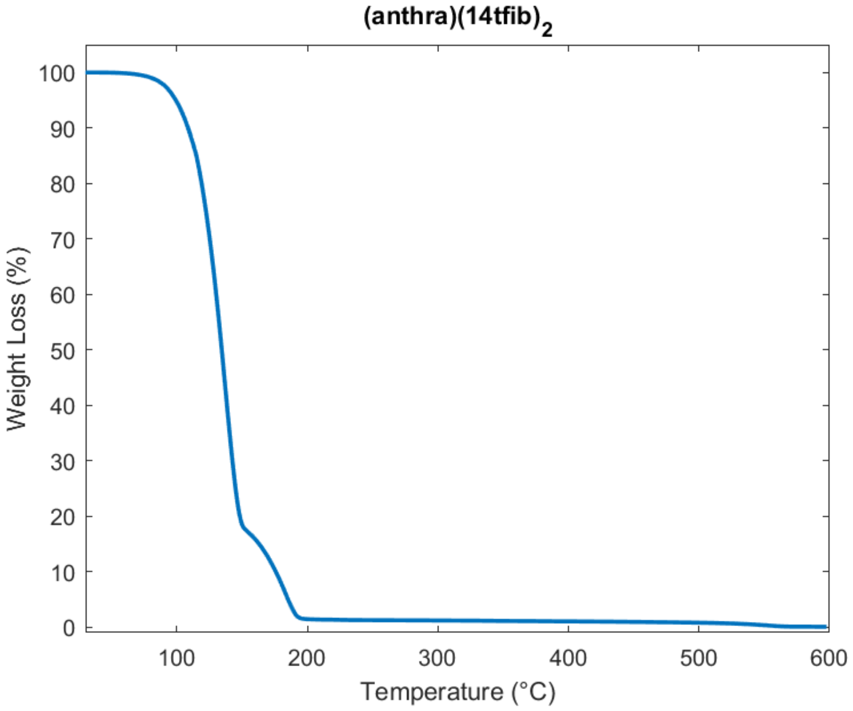

Supplementary Figure S24. TGA thermogram for (**anthra**)(**14tfib**)$_2$ cocrystal (30-600 °C, at a heating rate 10 °C min^{-1} in N_2 atmosphere).

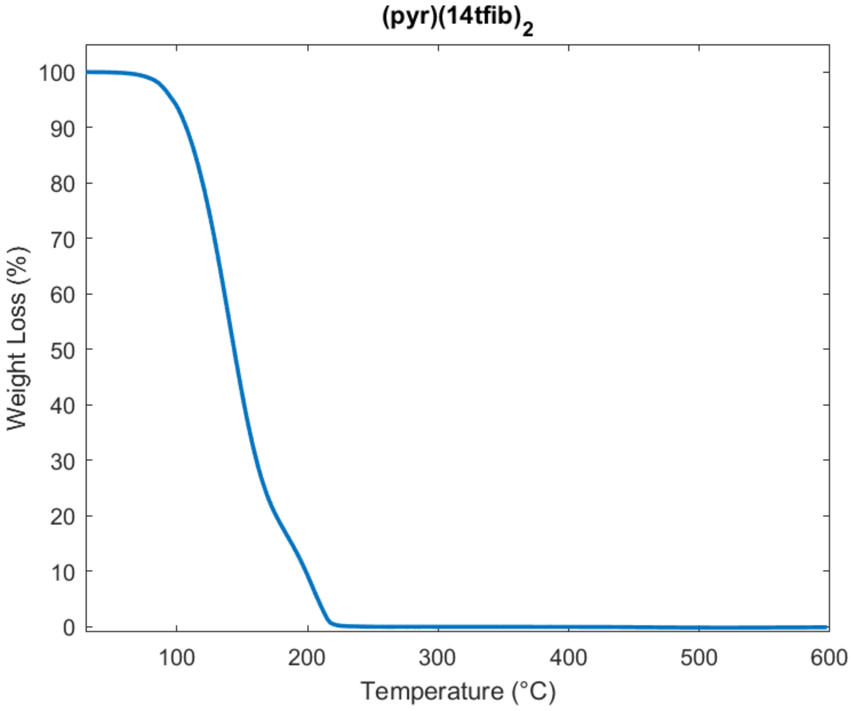

Supplementary Figure S25. TGA thermogram for (**pyr**)(**14tfib**)$_2$ cocrystal (30-600 °C, at a heating rate 10 °C min^{-1} in N_2 atmosphere).

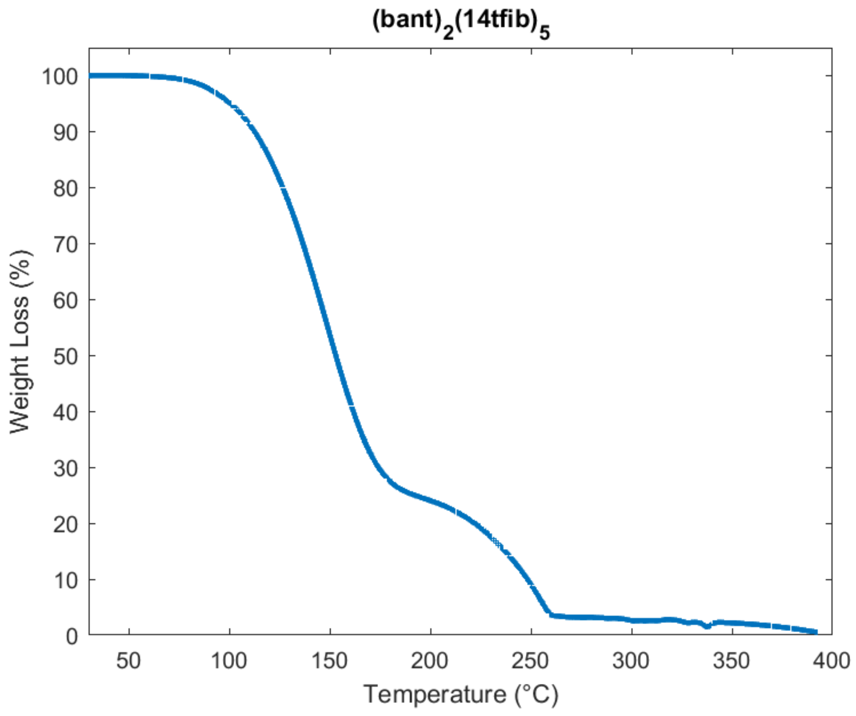

Supplementary Figure S26. TGA thermogram for (**bant**)$_2$(**14tfib**)$_5$ cocrystal (30-400 °C, at a heating rate 10 °C min^{-1} in air).

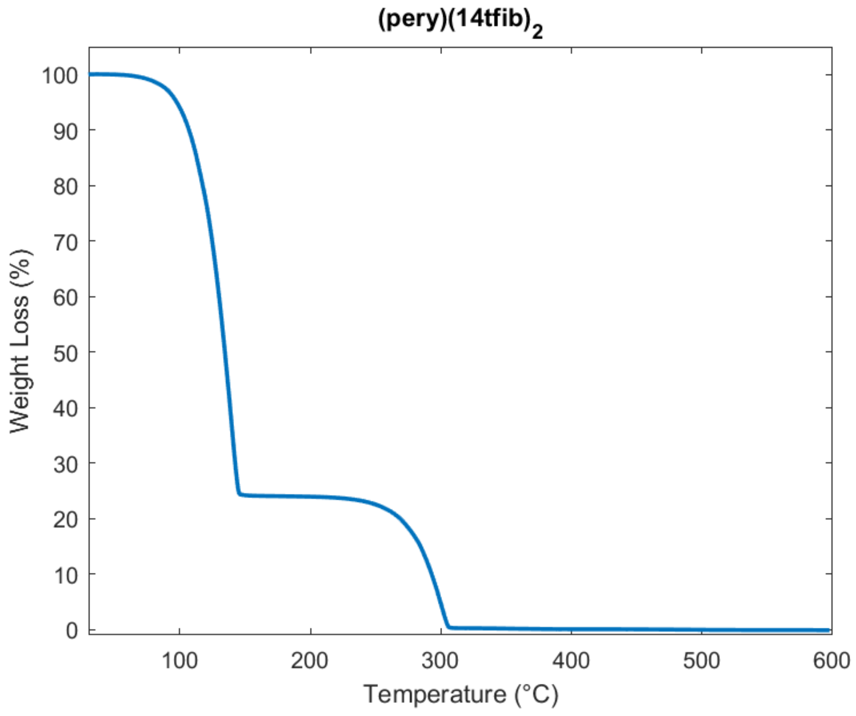

Supplementary Figure S27. TGA thermogram for (**pery**)(**14tfib**)$_2$ cocrystal (30-600 °C, at a heating rate 10 °C min^{-1} in N_2 atmosphere).

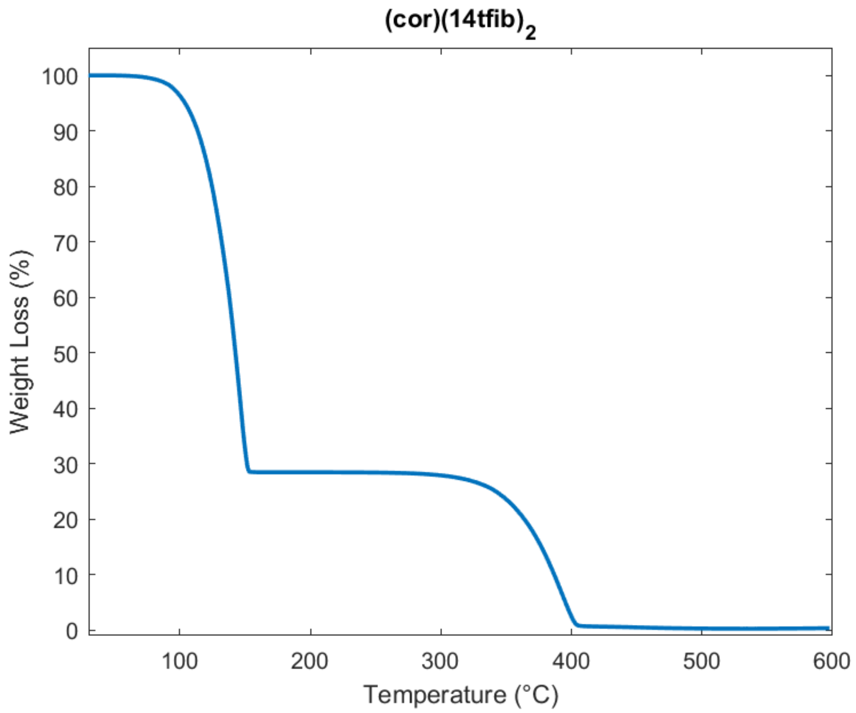

Supplementary Figure S28. TGA thermogram for (**cor**)(**14tfib**)$_2$ cocrsytal (30-600 °C, at a heating rate 10 °C min^{-1} in N_2 atmosphere).

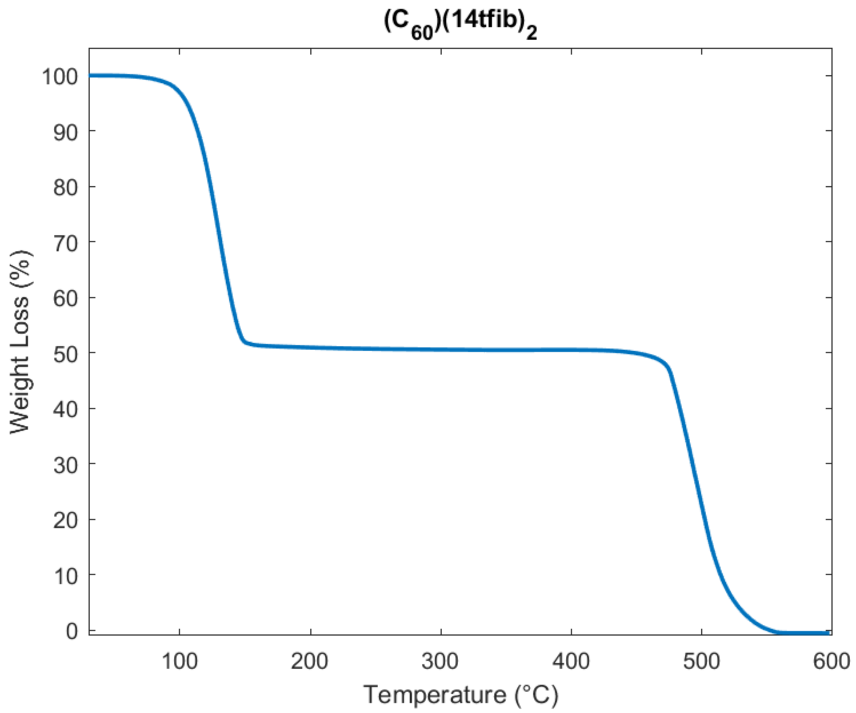

Supplementary Figure S29. TGA thermogram for (**C_{60}**)(**14tfib**)$_2$ cocrystal (30-600 °C, at a heating rate 10 °C min^{-1} in N_2 atmosphere).

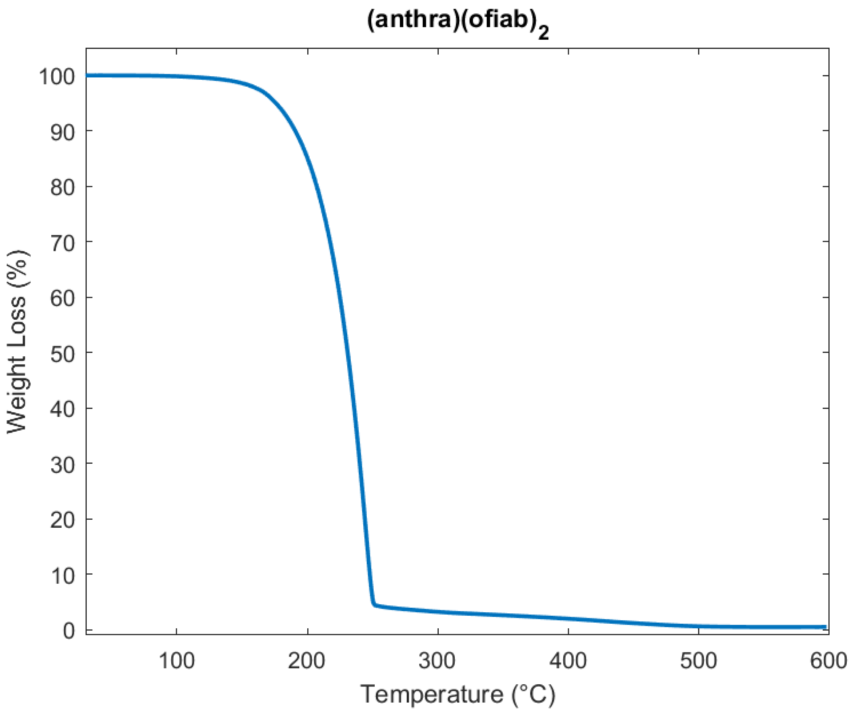

Supplementary Figure S30. TGA thermogram for (**anthra**)(**ofiab**)$_2$ cocrystal (30-600 °C, at a heating rate 10 °C min^{-1} in N_2 atmosphere).

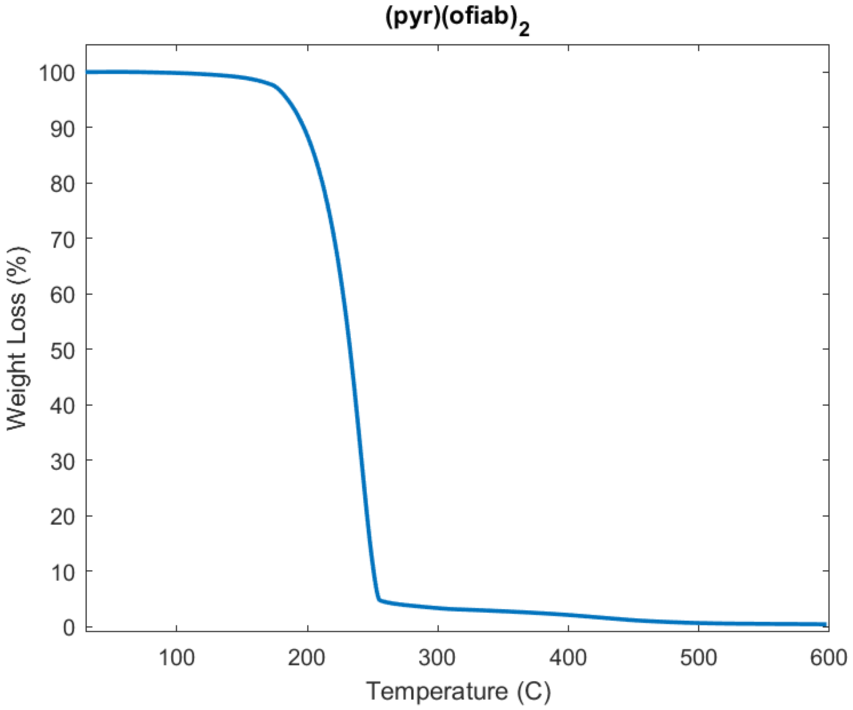

Supplementary Figure S31. TGA thermogram for (**pyr**)(**ofiab**)$_2$ cocrystal (30-600 °C, at a heating rate 10 °C min^{-1}, in N_2 atmosphere).

6. Analysis of the Cambridge Structural Database (CSD)

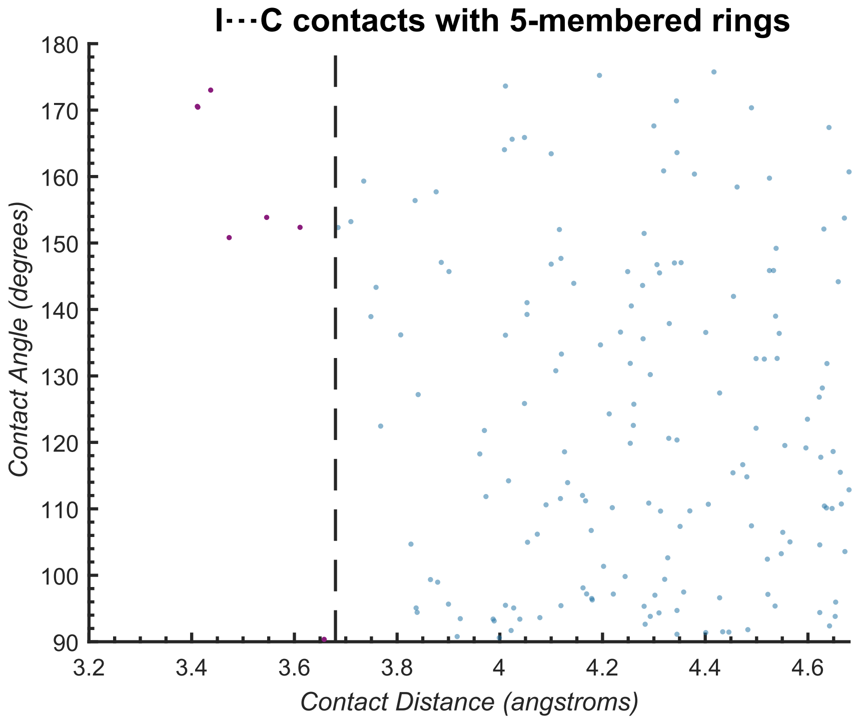

Supplementary Figure S32. Distribution of C-I···C contacts targeting a five-membered ring as the acceptor in the Cambridge Structural Database. I···C contact distances shorter than 3.68 Å are denoted in purple.

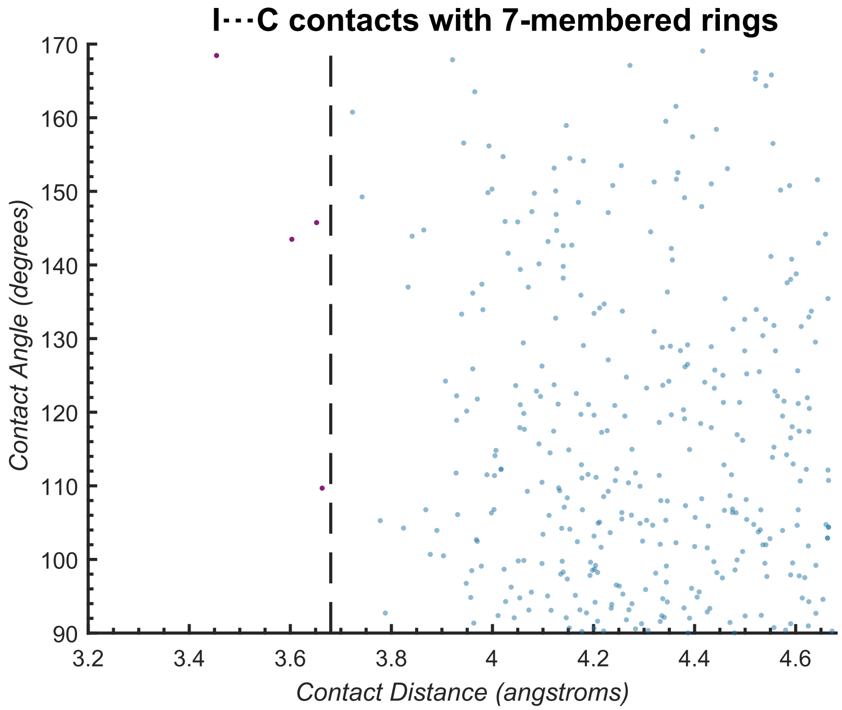

Supplementary Figure S33. Distribution of C-I···C contacts targeting a seven-membered ring as the acceptor in the Cambridge Structural Database. I···C contact distances shorter than 3.68 Å are denoted in purple.

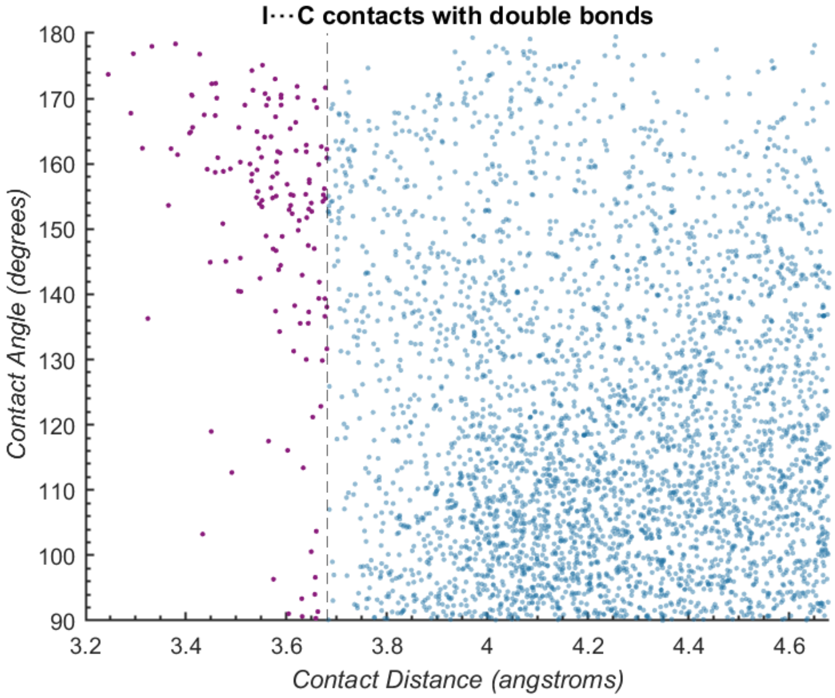

Supplementary Figure S34. Distribution of C-I···C contacts targeting double bond moieties as the acceptor in the Cambridge Structural Database. I···C contact distances shorter than 3.68 Å are denoted in purple.

7. FLUORESCENCE AND PHOSPHORESCENCE DATA

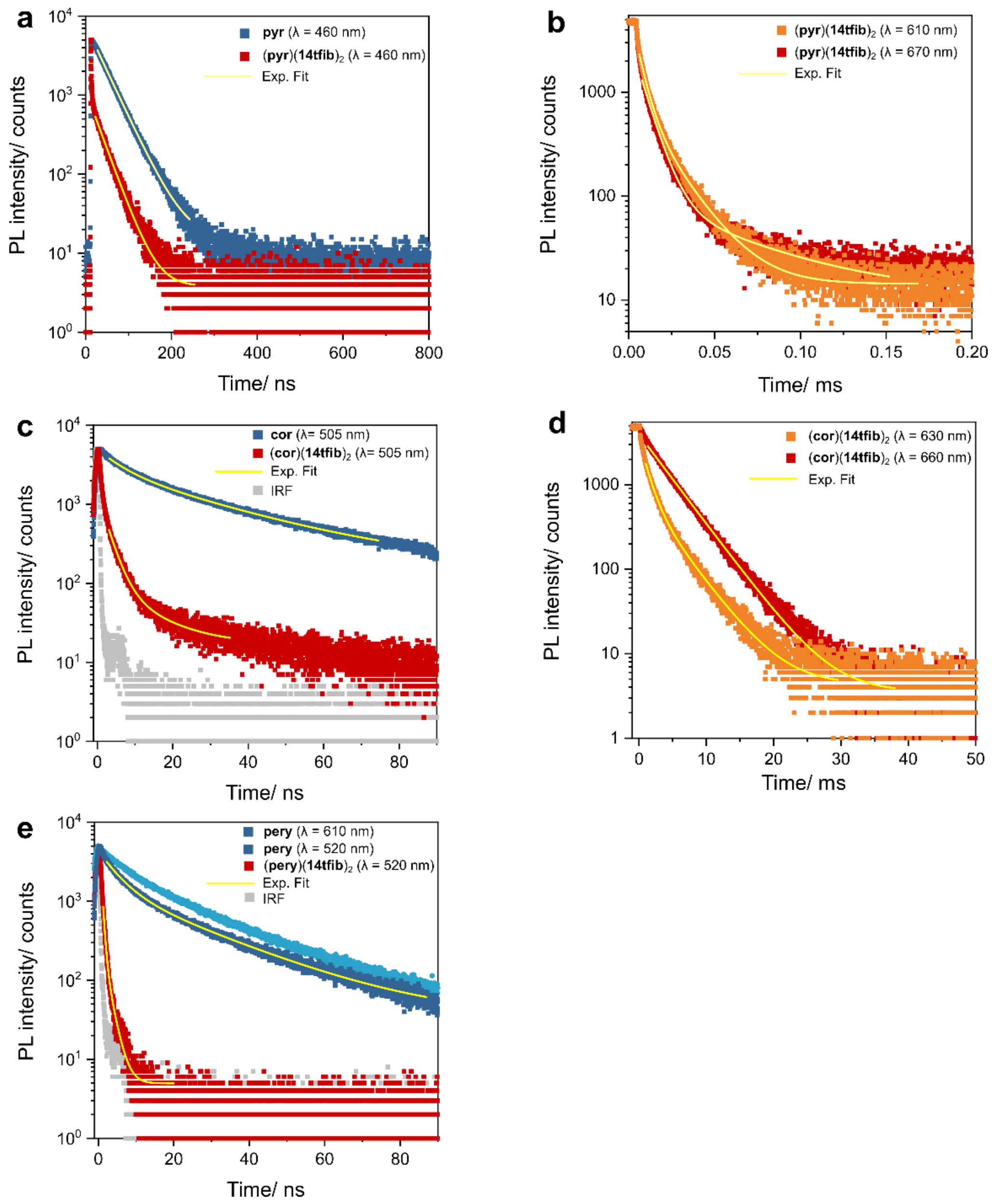

Supplementary Figure S35. Emission lifetime measurement plots, with corresponding exponential fitting for: a) **pyr** and (**pyr**)(**14tfib**)$_2$ at the emission wavelength of 460 nm; b) (**pyr**)(**14tfib**)$_2$ at emission wavelengths of 610 and 670 nm; c) **cor** and (**cor**)(**14tfib**)$_2$ at the emission wavelength of 505 nm; d) (**cor**)(**14tfib**)$_2$ at emission wavelengths of 630 nm and 660 nm; e) **pery** and (**pery**)(**14tfib**)$_2$ at emission wavelengths of 520 nm and 610 nm.

Supplementary Table S3. Fluorescence and phosphorescence lifetime data for **pyr**, **cor**, **pery** and the respective cocrystals based on the C-I···C_{π} halogen-bonded motif. Lifetimes are accompanied with associated fractional contributions to the average lifetime values.

**Material**	**Emission wavelength (in nm)**	**Emission lifetimes (τ)**	**Average lifetime (τ_{avg})**
**pyr**	460	37.3 ns (100%)	37.3 ns
(**pyr**)(**14tfib**)$_2$	460	24.4 ns (100%)	24.2 ns
	610	4.8 μs (73%), 17.4 μs (27%)	8.2 μs
	670	7.5 μs (93.7%), 52.9 μs (6.3%)	10.4 μs
**cor**	505	6.0 ns (42.6%), 28.3 ns (57.4%)	18.8 ns
(**cor**)(**14tfib**)$_2$	505	2.5 ns (90%), 10.6 ns (10%)	3.3 ns
	630	1.2 ms (64.4%), 4.1 ms (35.6%)	2.2 ms
	660	4.2 ms (100%)	4.2 ms
**pery**	520	4.7 ns (64.8%), 21.8 ns (35.2%)	10.7 ns
	610	7.1 ns (50%), 23.1 ns (50%)	15.1 ns
(**pery**)(**14tfib**)$_2$	520	0.5 ns (87.2%), 1.7 ns (12.8%)	< 0.62 ns

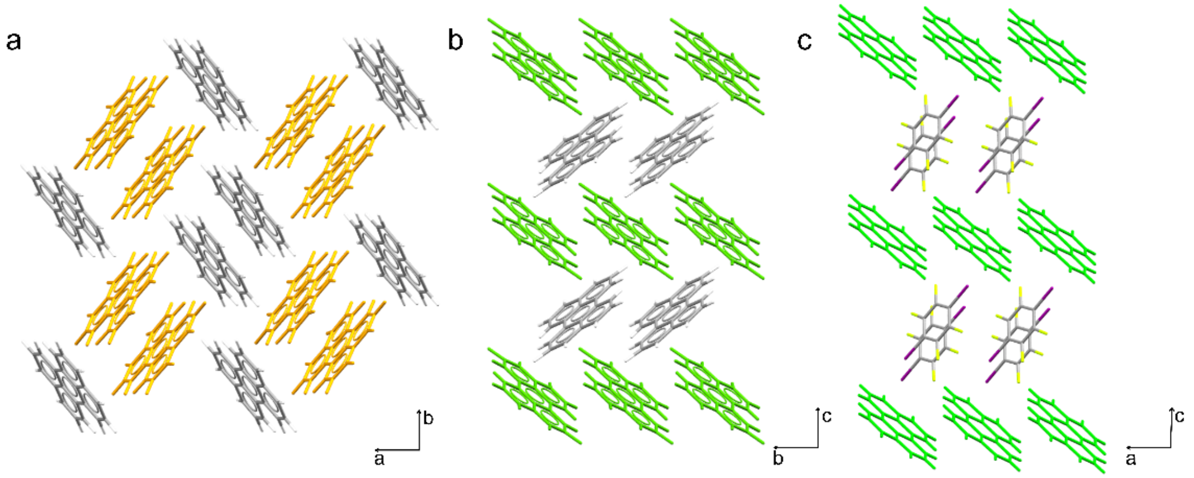

Supplementary Figure S36. Comparison of solid-state molecular packing in crystalline: a) α-**pery** (CSD code PERLEN05); b) β-**pery** (CSD code PERLEN07) and c) (**pery**)(**14tfib**)$_2$ cocrystal.

8. References

1. M. Zander and W. Franke, *Chem. Ber. Recl.*, 1958, **91**, 2794-2797.
2. A. Antoine John and Q. Lin, *J. Org. Chem.*, 2017, **82**, 9873-9876.
3. T. Friščić, S. L. Childs, S. A. A. Rizvi and W. Jones, *CrystEngComm*, 2009, **11**, 418-426.
4. T. Friščić, A. V. Trask, W. Jones and W. D. Motherwell, *Angew. Chem. Int. Ed. Engl.*, 2006, **45**, 7546-7550.
5. APEX3, SAINT and SADABS; Bruker AXS Inc.: Madison, Wisconsin, USA, 2016.
6. L. Krause, R. Herbst-Irmer, G. M. Sheldrick and D. Stalke, *J. Appl. Crystallogr.*, 2015, **48**, 3-10.
7. CrysAlisPRO, Oxford Diffraction /Agilent Technologies UK Ltd, Yarnton, England.
8. G. M. Sheldrick, *Acta Crystallogr. A Found. Adv.*, 2015, **71**, 3-8.
9. G. M. Sheldrick, *Acta Crystallogr. C Struct. Chem.*, 2015, **71**, 3-8.
10. O. V. Dolomanov, L. J. Bourhis, R. J. Gildea, J. A. K. Howard and H. Puschmann, *J. Appl. Crystallogr.*, 2009, **42**, 339-341.
11. C. B. Hubschle, G. M. Sheldrick and B. Dittrich, *J. Appl. Crystallogr.*, 2011, **44**, 1281-1284.
12. C. F. Macrae, I. J. Bruno, J. A. Chisholm, P. R. Edgington, P. McCabe, E. Pidcock, L. Rodriguez-Monge, R. Taylor, J. van de Streek and P. A. Wood, *J. Appl. Crystallogr.*, 2008, **41**, 466-470.
13. Persistence of Vision (TM). *Raytracer*. Persistence of Vision Pty. Ltd., Williamstown, 2004.
14. M. J. Frisch, *et al.* Gaussian 16, Revision C.01, Gaussian, Inc., Wallingford, CT, 2016.
15. C. Adamo and V. Barone, *J. Chem. Phys.*, 1999, **110**, 6158-6170.
16. T. H. Dunning, *J. Chem. Phys.*, 1989, **90**, 1007-1023.
17. K. A. Peterson, D. Figgen, E. Goll, H. Stoll and M. Dolg, *J. Chem. Phys.*, 2003, **119**, 11113-11123.
18. F. Weigend and R. Ahlrichs, *Phys. Chem. Chem. Phys.*, 2005, **7**, 3297-3305.
19. B. P. Pritchard, D. Altarawy, B. Didier, T. D. Gibson and T. L. Windus, *J. Chem. Inf. Model.*, 2019, **59**, 4814-4820.
20. K. Momma and F. Izumi, *J. Appl. Crystallogr.*, 2011, **44**, 1272-1276.
21. S. J. Clark, M. D. Segall, C. J. Pickard, P. J. Hasnip, M. J. Probert, K. Refson and M. C. Payne, *Z. Kristallogr. Cryst. Mater.*, 2005, **220**, 567-570.
22. T. Björkman, *Comput. Phys. Commun.*, 2011, **182**, 1183-1186.
23. J. P. Perdew, K. Burke and M. Ernzerhof, *Phys. Rev. Lett.*, 1996, **77**, 3865-3868.
24. S. Ehrlich, J. Moellmann, W. Reckien, T. Bredow and S. Grimme, *ChemPhysChem*, 2011, **12**, 3414-3420.
25. I. J. Bruno, J. C. Cole, P. R. Edgington, M. Kessler, C. F. Macrae, P. McCabe, J. Pearson and R. Taylor, *Acta Crystallogr. B*, 2002, **58**, 389-397.
26. M. Mantina, A. C. Chamberlin, R. Valero, C. J. Cramer and D. G. Truhlar, *J. Phys. Chem. A*, 2009, **113**, 5806-5812.
27. Haynes, W. M. *CRC Handbook of Chemistry and Physics*; CRC Press, 2014.
28. V. Nemec and D. Cinčić, *CrystEngComm*, 2016, **18**, 7425-7429.
29. O. S. Bushuyev, T. C. Corkery, C. J. Barrett and T. Friščić, *Chem. Sci.* 2014, **5**, 3158-3164.
